# A sensitivity analysis approach for informative dropout using shared parameter models

**DOI:** 10.1111/biom.13027

**Published:** 2019-04-01

**Authors:** Li Su, Qiuju Li, Jessica K. Barrett, Michael J. Daniels

**Affiliations:** ^1^ MRC Biostatistics Unit School of Clinical Medicine, University of Cambridge Cambridge CB2 0SR U.K.; ^2^ Department of Statistics University of Florida Gainesville Florida 32611

**Keywords:** Bayesian inference, Joint models, longitudinal data, missing data, random effects

## Abstract

Shared parameter models (SPMs) are a useful approach to addressing bias from informative dropout in longitudinal studies. In SPMs it is typically assumed that the longitudinal outcome process and the dropout time are independent, given random effects and observed covariates. However, this conditional independence assumption is unverifiable. Currently, sensitivity analysis strategies for this unverifiable assumption of SPMs are underdeveloped. In principle, parameters that can and cannot be identified by the observed data should be clearly separated in sensitivity analyses, and sensitivity parameters should not influence the model fit to the observed data. For SPMs this is difficult because it is not clear how to separate the observed data likelihood from the distribution of the missing data given the observed data (i.e., ‘extrapolation distribution’). In this article, we propose a new approach for transparent sensitivity analyses for informative dropout that separates the observed data likelihood and the extrapolation distribution, using a typical SPM as a working model for the complete data generating mechanism. For this model, the default extrapolation distribution is a skew‐normal distribution (i.e., it is available in a closed form). We propose anchoring the sensitivity analysis on the default extrapolation distribution under the specified SPM and calibrate the sensitivity parameters using the observed data for subjects who drop out. The proposed approach is used to address informative dropout in the HIV Epidemiology Research Study.

## 1 Introduction

### 1.1 Shared parameter models and sensitivity analysis strategies

Shared parameter models (SPMs) are one of the three main model‐based approaches to dealing with informative dropout in longitudinal studies, where ‘informative’ means that the dropout process depends on the unobserved outcomes even after conditioning on the observed data (Tsiatis and Davidian, 2004; Daniels and Hogan, 2008). In SPMs the dependence between the longitudinal outcome process and the dropout process is often characterized by a set of time‐invariant random effects. For example, a popular parameterization is to specify simple random intercepts and random time slopes in the longitudinal outcome model, while they are also included in the dropout model as covariates. Given the random effects and observed covariates, it is typically assumed that the longitudinal outcome process (i.e., the *complete* longitudinal outcome data that are intended to be collected) and the dropout time process are independent. This conditional independence assumption can be classified as a *latent ignorability* assumption discussed in Harel and Schafer (2009). However, it is unverifiable because it is not possible to assess the conditional independence between the unobserved outcomes after dropout and the dropout time. Therefore, sensitivity analyses are required for SPMs. In this article we focus on the sensitivity of the inference for marginal covariate effects on the longitudinal outcome to the unverifiable assumption of SPMs.

Unfortunately, unlike pattern mixture models (PMMs), research for sensitivity analysis strategies based on SPMs is very limited. Sensitivity analyses, as defined in Daniels and Hogan (2008), have been done for SPMs in a series of articles by Creemers and colleagues 2010, 2011. Creemers et al. (2010) introduce a generalized class of SPMs by incorporating additional random effects (not typically found in the original SPM) as sensitivity parameters that connect the conditional distribution of the missing data given the observed data (i.e., the extrapolation distribution) and the model for missingness indicators. The corresponding sensitivity parameters are not easily interpretable. Creemers et al. (2011) also use the generalized class of SPMs with additional random effects, but their approach is more similar to what we propose here because identifying restrictions like the missing at random (MAR) assumption or the non‐future dependence assumption 2003 are used to define sub‐classes of the generalized SPM that satisfy these restrictions. However, in this article we advocate using the typical SPM with the conditional independence assumption and its default extrapolation distribution as the basis of a sensitivity analysis (i.e., there are no additional random effects specified to link the the extrapolation distribution and the dropout process) and introduce sensitivity parameters that are easily interpretable in the context of the typical SPM.

Following the principle of a transparent sensitivity analysis advocated by Daniels and Hogan (2008), we propose a new sensitivity analysis approach for informative dropout based on a typical SPM with the conditional independence assumption, where the likelihood for observed data and the sensitivity parameter are clearly separated. Within the Bayesian framework, we first fit the SPM proposed by Barrett et al. (2015) to the observed longitudinal outcome data and the dropout time. Specifically, a linear mixed model is assumed for the complete longitudinal outcomes, while the dropout time distribution follows a probit model for the discrete hazard of dropout. The two models are linked by correlated normal random effects. Given these random effects and observed covariates, the longitudinal outcome process and the dropout process are assumed to be independent. We show that under this SPM, the default extrapolation distribution for missing data after dropout is a skew‐normal distribution depending on model parameters, covariates and observed longitudinal outcome data. The proposed sensitivity analysis is then anchored at this ‘default’ extrapolation distribution and a piece‐wise linear model for individual longitudinal profiles is specified to determine the extrapolation distribution at a fixed value of a global sensitivity parameter. The global sensitivity parameter can be interpreted as the parameter that controls the overall deviation of the individual longitudinal profiles after dropout from the default extrapolations under the SPM. Given a specific set of values for the covariates, posterior samples of the model parameters and an informative prior for the global sensitivity parameter based on the substantive context, we use G‐computation 1986, 2014 to obtain the inferences for the marginal (population‐averaged) covariate effects on the longitudinal outcome under both the default extrapolation distribution of the SPM and the extrapolation distribution specified in the sensitivity analysis. The G‐computation and the Markov Chain Monte Carlo (MCMC) for fitting the SPM are separate; therefore our sensitivity analysis approach does not impact the fit of the model to the observed data.

### 1.2 Motivating example

This work is motivated by data from the HIV Epidemiology Research Study (HERS). The HERS was a longitudinal study of 1310 women with, or at high risk for, HIV infection from 1993 to 2000 2003. During the study 12 visits were scheduled, where a variety of clinical, behavioral and sociological outcomes were recorded approximately every 6 months. We will focus on the 850 women who were HIV‐positive and had CD4 count measurements at enrollment.

Like many other long‐term follow‐up studies, attrition by dropout in the HERS is substantial, with more than half of the women not completing the study. Moreover, as suggested by previous analyses of these data (Hogan et al., 2004; Daniels and Hogan, 2008), dropout was likely informative and related to the disease progression characterized by CD4 counts. In other words, the unobserved CD4 counts among those who dropped out could be systematically lower than those who continued follow‐up, even after adjusting for covariates and observed CD4 counts. Hogan et al. (2004) adopted the pattern mixture modeling approach to dealing with this informative dropout problem when estimating the marginal effects of baseline covariates (HIV viral load, HIV symptom severity, antiretroviral treatment status) on the longitudinal CD4 count for the HERS data. In this article, we choose the shared parameter modeling approach for the HERS data and implement the proposed sensitivity analysis strategy tailored to SPMs. Because HIV disease progression, represented by changes in CD4 count, is believed to be strongly associated with the dropout, we use random effects in the model for CD4 counts to characterize the HIV disease progression. These random effects also govern the relationship between HIV disease progression and dropout.

The rest of the article is organized as follows. In Section 2, we describe the proposed sensitivity analysis strategy, show its implementation using the specified SPM and derive the default extrapolation distribution for the missing outcome under this SPM. In Section 3, the HERS data are analyzed to illustrate the proposed methods. We conclude with a discussion in Section 4.

## 2 Methods

### 2.1 Sensitivity analysis strategy

**Figure 1:**
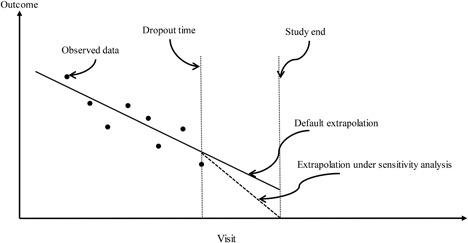
Graphical illustration of the default extrapolation under a typical SPM and the possible extrapolation under the proposed sensitivity analysis.

In this section, we propose a general sensitivity analysis strategy for informative dropout using SPMs. Because random effects are often used to characterize the individual longitudinal profile, we can interpret the default extrapolation under a SPM as trying to use the same random effect distribution given observed data *before dropout* for characterizing the individual longitudinal profile *after dropout*. However, this might not be true if this individual longitudinal profile beyond dropout varies from what the SPM predicts under the conditional independence assumption. For example, in the HERS example, it is plausible that the unobserved CD4 counts after patients’ dropout were decreasing more rapidly than the SPM predicts. Therefore the individual longitudinal profile after dropout might not be able to be described by the conditional distribution of the random effects given all observed data. This discrepancy cannot be identified from the observed data, and can be the basis for a sensitivity analysis. Figure 1 provides a graphical illustration for the default extrapolation under a SPM and the possible extrapolation under our proposed sensitivity analysis strategy.

We propose to anchor the sensitivity analysis at the default extrapolation distribution of the SPM. In the next sections, we describe a typical SPM in our approach and the details of the sensitivity analysis strategy for it.

### 2.2 Model

Suppose that *N* independent patients are followed up over time. For the *i*‐th (i=1,…,N) patient, longitudinal measurements $Y_{i}={\left(Y_{i1},\ldots ,Y_{\mathrm{iM}}\right)}^{T}$ are scheduled to be taken at time points $t_{i1},\ldots ,t_{\mathrm{iM}}$ in [0,T], where *T* is the total length of scheduled follow‐up in the study. However, patient can withdraw from the study during the follow‐up, which terminates the observation of the longitudinal outcome $Y_{i}$. Let $S_{i}$ denote the ‘dropout time’ for the *i*‐th patient. Information about exact time of dropout is often not available in practice. Therefore, we define $S_{i}$ to be the number of the last follow‐up visit, and hence it is discrete. When $S_{i}=j-1<M$ (j=2,…,M), the outcome vector ${\left(Y_{\mathrm{ij}},\ldots ,Y_{\mathrm{iM}}\right)}^{T}$ are unobserved. If the patient has complete data, then $S_{i}$ is treated as administratively censored at visit *M*. We let $Y_{i}^{o}={\left(Y_{i1},\ldots ,Y_{i,j-1}\right)}^{T}$ denote the vector of the observed outcomes and assume that $Y_{i1}$ is always observed (baseline outcome).

In this article, we adopt the SPM in Barrett et al. (2015) that is well suited to the HERS data. First, the *complete* outcome $Y_{\mathrm{ij}}$ (j=1,…,M) at visit *j* is assumed to follow

(1) $$Y_{\mathrm{ij}}=x_{\mathrm{ij}}^{T}\beta +z_{\mathrm{ij}}^{T}b_{i}+\varepsilon_{\mathrm{ij}},$$

where β is a p×1 vector of regression coefficients associated with exogenous covariates $x_{\mathrm{ij}}$ (fixed effects), $b_{i}$ is a q×1 vector of random effects that are associated with covariates $z_{\mathrm{ij}}$, $\varepsilon_{\mathrm{ij}}$ is the measurement error that is independent of covariates $x_{\mathrm{ij}}$ and $z_{\mathrm{ij}}$, and ${\left(\varepsilon_{i1},\ldots ,\varepsilon_{\mathrm{iM}}\right)}^{T}∼N(0,\sigma_{\varepsilon }^{2}I_{M\times M})$. The covariate vectors $x_{\mathrm{ij}}$ and $z_{\mathrm{ij}}$ are assumed to be completely observed. In the HERS application, we assume $x_{\mathrm{ij}}$ includes ${\left(1,t_{\mathrm{ij}}\right)}^{T}$ and baseline covariates; and $z_{\mathrm{ij}}={\left(1,t_{\mathrm{ij}}\right)}^{T}$, so $b_{i}$ corresponds to a random intercept and a random slope. The random effects $b_{i}$ follow a multivariate normal distribution $N(0,{𝚺}_{b})$.

Let $\lambda_{i,j-1}=P(S_{i}=j-1|S_{i}\geq j-1,b_{i},x_{S,i,j-1},W_{i,j-1})$ be the discrete hazard of dropout at visit j−1 (j=2,…,M). We assume a probit model,

(2) $$\lambda_{i,j-1}=1-\Phi \left\{x_{S,i,j-1}^{T}\alpha +{\left(W_{i,j-1}b_{i}\right)}^{T}\gamma_{j-1}\right\},$$

where Φ(·) is the standard normal cumulative distribution function, $x_{S,i,j-1}$ is a $p_{S}\times 1$ vector of covariates (possibly time‐varying) with regression coefficients α. $W_{i,j-1}$ is a matrix for constructing a $q_{S}\times 1$ vector of linear combinations of $b_{i}$. In the HERS application, we have $W_{i,j-1}=I_{2\times 2}$ and $q_{S}=2$. Other examples of $W_{i,j-1}$ include ${\left(1,t_{i,j-1}\right)}^{T}$; see discussion on these parameterizations in Chapter 7 of Rizopoulos (2012). $\gamma_{j-1}$ is an association parameter vector that relates the longitudinal outcome and the dropout time via the random effects $b_{i}$. Note that if $\gamma_{j-1}=0$ then the dropout is ignorable. Given $b_{i}$ and the covariates, the *complete* longitudinal outcome $Y_{i}$ and the dropout time $S_{i}$ are assumed to be independent.

### 2.3 Estimation and inference

We use a Bayesian approach for estimation and inference of the SPM. For simplicity of presentation we suppress the conditioning on $x_{\mathrm{ij}}$, $z_{\mathrm{ij}}$, $x_{S,i,j-1}$ and $W_{i,j-1}$. The observed data are $\left\{Y_{i}^{o},S_{i}=j-1\right\}$ (i=1,…,N), and the observed data likelihood contribution from the *i*‐th patient given the random effects is

(3) $$\begin{array}{ccc} L_{i}(\theta |Y_{i}^{o},S_{i} & = & j-1,b_{i})=f(Y_{i}^{o}|b_{i};\theta )\\ f(S_{i} & = & j-1|b_{i};\theta )f(b_{i};\theta ), \end{array}$$

where θ denotes all unknown parameters in the SPM that include regression coefficients β, α, $\gamma_{j-1}$ and covariance parameters in ${𝚺}_{b}$. Let $X_{i}={\left(x_{i1},\ldots ,x_{{\mathrm{iS}}_{i}}\right)}^{T}$ and $Z_{i}={\left(z_{i1},\ldots ,z_{{\mathrm{iS}}_{i}}\right)}^{T}$. The likelihood from the observed longitudinal outcome given the random effects is

$$\begin{array}{ccc} f(Y_{i}^{o}|b_{i};\theta ) & = & \text{exp}\{-\text{log}(2\pi )S_{i}/2\\ & & -\text{log}(|V_{i}|)/2-{\left(Y_{i}^{o}-\mu_{i}\right)}^{T}V_{i}^{-1}(Y_{i}^{o}-\mu_{i})/2\}, \end{array}$$

where $\mu_{i}=X_{i}\beta +Z_{i}b_{i}$ and $V_{i}=\sigma_{\varepsilon }^{2}I_{S_{i}\times S_{i}}$. The observed data likelihood contribution from the dropout time given the random effects is

(4) $$\begin{array}{ccc} f(S_{i} & = & j-1|b_{i};\theta )\\ & = & \left\{\begin{array}{ccc} \lambda_{i1} & \mathrm{when} & j-1=1\\ \lambda_{i,j-1}\prod_{l=1}^{j-2}(1-\lambda_{\mathrm{il}}) & \mathrm{when} & 1<j-1<M\\ \prod_{l=1}^{j-2}(1-\lambda_{\mathrm{il}}) & \mathrm{when} & j-1=M \end{array}\right. \end{array}$$

Recall the density $f(b_{i};\theta )$ is $N(0,{𝚺}_{b})$. We follow Daniels and Zhao (2003) and use the modified Cholesky decomposition to parameterize ${𝚺}_{b}$ such that positive definiteness is guaranteed for ${𝚺}_{b}$. In the HERS analysis in Section 3, we assume $b_{i}={\left(b_{i1},b_{i2}\right)}^{T}$, where $b_{i1}$ is a random intercept and $b_{i2}$ is a random slope. Then $b_{i}$ can be written in two parts: $b_{i1}=e_{i1}$, $b_{i2}=\delta b_{i1}+e_{i2}$. The first equation corresponds to the marginal distribution of the random intercept, and the second equation describes the conditional distribution of the random slope given the random intercept. Let $\sigma_{1}^{2}$ and $\sigma_{2}^{2}$ be the variances of $e_{i1}$ and $e_{i2}$, respectively. Then the covariance matrix ${𝚺}_{b}$ can be written as

$${𝚺}_{b}=\left[\begin{array}{cc} \sigma_{1}^{2} & \delta \sigma_{1}^{2}\\ \delta \sigma_{1}^{2} & \delta^{2}\sigma_{1}^{2}+\sigma_{2}^{2} \end{array}\right].$$

We provide details of the prior specification and posterior inference in the context of the HERS analysis in Section 3.

### 2.4 Default extrapolation distribution under the SPM

To derive the default extrapolation distribution of the missing outcome $Y_{\mathrm{ik}}$ (k=j,…,M) after dropout at visit j−1, we first need to derive the conditional distribution of the random effects $b_{i}$, given the observed data $Y_{i1},\ldots ,Y_{i,j-1}$, $S_{i}=j-1$, and $H_{i,j-1}$. Here $H_{i,j-1}$ is the collection of the history of the corresponding covariates ${\bar{x}}_{i,j-1}$, ${\bar{z}}_{i,j-1}$, ${\bar{x}}_{S,i,j-1}$, ${\bar{W}}_{i,j-1}$ up to visit j−1 (an overbar represents the history of a process). The conditional density of $b_{i}$ given the observed data is

(5) $$\begin{array}{ccc} & & f(b_{i}|Y_{i1},\ldots ,Y_{i,j-1},S_{i}=j-1,H_{i,j-1})\\ & \propto & f(b_{i};\theta )f(Y_{i1},\ldots ,Y_{i,j-1}|H_{i,j-1},b_{i};\theta )\lambda_{i,j-1}\prod_{l=1}^{j-2}(1-\lambda_{\mathrm{il}}). \end{array}$$

This conditional density is a member of the class of multivariate skew‐normal distribution described in González‐Farías et al. (2004) and Arnold (2009). Details of the proof for this distribution can be found in supporting information.

Recall that the missing outcome $Y_{\mathrm{ik}}$ (k=j,…,M) after dropping out at visit j−1 is assumed to follow the regression model of the form $Y_{\mathrm{ik}}=x_{\mathrm{ik}}^{T}\beta +z_{\mathrm{ik}}^{T}b_{i}+\varepsilon_{\mathrm{ik}}$ in (1) with the error term assumed to be independent of the random effects and covariates. Given the additive property of the multivariate skew‐normal distribution 2004, the conditional distribution of $Y_{\mathrm{ik}}$ given the observed data, $Y_{i1},\ldots ,Y_{i,j-1}$, $S_{i}$, $H_{\mathrm{ik}}$, $x_{\mathrm{ik}}$ and $z_{\mathrm{ik}}$, can also be shown to follow a skew‐normal distribution; see details in supporting information. This conditional distribution for $Y_{\mathrm{ik}}$ is the default extrapolation distribution under the specified SPM. Given the model parameters and covariates, sampling from this extrapolation distribution can proceed by separately drawing from the conditional distribution of $b_{i}$ given the observed data and from the distribution of $\varepsilon_{\mathrm{ik}}$ and then computing $x_{\mathrm{ik}}^{T}\beta +z_{\mathrm{ik}}^{T}b_{i}+\varepsilon_{\mathrm{ik}}$.

### 2.5 Sensitivity analysis for the SPM

Without loss of generality, we let $z_{\mathrm{ik}}={\left(1,t_{\mathrm{ik}}\right)}^{T}$ and then $b_{i}={\left(b_{i1},b_{i2}\right)}^{T}$ represents the random intercept and random slope. In the sensitivity analysis, the model for $Y_{\mathrm{ik}}$ (k=j,…,M) after dropout at visit j−1 is assumed to follow a piece‐wise linear model

(6) $$\begin{array}{ccc} & & Y_{\mathrm{ik}}=x_{\mathrm{ik}}^{T}\beta +b_{i1}+b_{i2}t_{\mathrm{ik}}+\Delta_{i}{\left(t_{\mathrm{ik}}-t_{i,j-1}\right)}_{+}+\varepsilon_{\mathrm{ik}}, \end{array}$$

where ${\left(x\right)}_{+}=x$ if x>0 and 0 otherwise. Note that ${\left(b_{i1},b_{i2}\right)}^{T}$ in (6) follows the distribution in (5). $\Delta_{i}$ is the change of the slope for the *i*‐th patient after dropout at visit j−1 (i.e., deviation from the random slope $b_{i2}$; see Figure 1), which can depend on the observed data of the *i*‐th patient; when $\Delta_{i}=0$ for all *i* we obtain the default extrapolation distribution. For example, let

(7) $$\begin{array}{ccc} \Delta_{i} & = & a\left\{\left(M-S_{i})/(M-1\right)\right\}\sigma_{b_{i2}}, \end{array}$$

where *a* is the sensitivity parameter and $\sigma_{b_{i2}}={\left\{\mathrm{Var}(b_{i2}|Y_{i1},\ldots ,Y_{i,j-1},S_{i}=j-1,H_{i,j-1})\right\}}^{1/2}$ is the standard deviation of the random slope given the observed data of the *i*‐th patient. When $S_{i}=M$, the patient has complete data, therefore no adjustment for the slope $b_{i2}$ is made and $\Delta_{i}=0$. $\Delta_{i}$ is proportional to $\left(M-S_{i})/(M-1\right)$, which allows more adjustment of the random slope made for earlier dropout because these patients might have more severe disease progression than what is characterized by the random effects. In particular, when $S_{i}=1$ and the patient drops out right after baseline, the adjustment is the largest with $\Delta_{i}=a\sigma_{b_{i2}}$, i.e., *a* times standard deviation of the random slope given the observed data of the *i*‐th patient. If $S_{i}=M-1$ and the patient almost completes the study except for the last scheduled visit, the adjustment is only a/(M−1) times standard deviation of the random slope given the observed data. We specify $\Delta_{i}$ to be proportional to $\sigma_{b_{i2}}$ to allow for the adjustment calibrated to the observed outcome variation given the individual characteristics of a specific patient. Note that $\Delta_{i}$ implicitly depends on the covariates because $\sigma_{b_{i2}}$ is the posterior standard deviation of the random slope conditional on all observed data (including the covariates). Therefore, implicitly the approach allows interactions between $\Delta_{i}$ and the covariates. Finally, *a* is a single sensitivity parameter that controls the overall deviation of the individual longitudinal profiles after dropout from the default extrapolations under the SPM for the study sub‐population with dropout.

Within the Bayesian framework, we can specify a prior for *a*. For example, in the HERS example in Section 3, we believe that patients can have more rapidly decreasing CD4 count profiles after dropout, therefore *a* is assumed to follow a triangular distribution with the range [−2,0] and the mode at −1. Thus we expect at most a two‐standard‐deviation downward change for the slope for the earliest dropouts and overall the change is centered at one standard deviation. When possible, the prior for the global sensitivity parameter *a* should be elicited from expert opinion (or historical information).

Sampling from the extrapolation distribution in the sensitivity analysis requires calculation of $\sigma_{b_{i2}}$. In supporting information, we show that this standard deviation is a function of the model parameters and observed data. We then calculate $\Delta_{i}$ in (7), given the sensitivity parameter, and use the model in (6) to sample from the extrapolation distribution.

To assess the impact of the sensitivity parameter on the final inference, we use Monte Carlo integration (i.e., G‐computation) to calculate the predicted means of the longitudinal outcome and summarize the marginal covariate effects on these predicted means for both the fitted SPM and sensitivity analysis. Specifically, the steps are:

1. Draw a sample from the prior for the sensitivity parameter *a*.
2. Draw a sample of $\left(Y_{i},S_{i}\right)$ based on the specified SPM, a specific set of covariate values, and a single set of posterior samples of the model parameters.
3. $Y_{i}$ is truncated at $S_{i}$ to obtain the replicated observed longitudinal data vector $Y_{i}^{o}$.
4. If $S_{i}<M$, then sample the missing outcomes from the default extrapolation distribution under the SPM and from the extrapolation distribution based on the model (6) and the current sample of *a*.
5. Repeat Steps 2–4 for 100N times. Note that the size of the Monte Carlo samples needs to be large relative to the sample size *N*. Here we follow Linero and Daniels (2018) and use 100 times the sample size.
6. Calculate summaries of all longitudinal outcome samples, e.g., average changes of longitudinal outcomes from baseline to specific follow‐up visits.
7. Repeat Steps 2–6 for other sets of covariate values and calculate baseline covariate effects on the longitudinal data summaries in Step 6 using contrasts between covariate groups.
8. Repeat Steps 1–7 for the entire set of posterior samples of model parameters, and summarize the posterior distribution of the baseline covariate effects obtained in Step 7.

## 3 Application to the HERS data

In this section, we implement the proposed approach to the HERS data. Of the 850 women who were HIV‐positive and had CD4 count data at baseline, we exclude 23 women from the analysis because their baseline covariate data were missing. The dropout time is treated as discrete and set as the number of the last follow‐up visit. For those women who finished 12 scheduled visits, their dropout times are treated as administratively censored at visit 12. During the follow‐up, 566 (7.6%) CD4 count measurements were intermittently missing before the patients’ dropout or the end of study. We assume that this intermittent missingness is latent ignorable 2009. That is, given the observed outcomes, random effects, dropout time, and covariates, the intermittent missingness is ignorable.

### 3.1 Fitted model

Following the previous analysis of the HERS data 2004, we assume a linear mixed model for the *complete* longitudinal measurements of CD4 count as follows,

(8) $$Y_{\mathrm{ij}}=x_{\mathrm{ij}}^{T}\beta +b_{i1}+b_{i2}j^{*}+\varepsilon_{\mathrm{ij}},$$

where $Y_{\mathrm{ij}}$ is the square root of CD4 count at visit *j* after standardization by taking (y−18)/7 and $x_{\mathrm{ij}}$ is the covariate vector, including the visit $j^{*}=(j-1)/11$, indicator variables for HIV viral load group (0,500], (500,5000], (5000,30,000] (copies/ml) at baseline, indicator of antiretroviral therapy (ART) at baseline, HIV symptomatology (presence of HIV‐related symptoms on a scale from 0 to 5) at baseline and the interactions between time (visit) and these baseline covariates. $b_{i1}$ and $b_{i2}$ are random intercept and slope, respectively, and they follow the multivariate normal distribution with mean zero and covariance ${𝚺}_{b}$, as parameterized by the modified Cholesky decomposition. The error term follows $\varepsilon_{\mathrm{ij}}\overset{i.i.d.}{∼}N(0,\sigma_{\varepsilon }^{2})$, which is independent of the random effects.

Based on some preliminary data exploration, we specify the following probit model for the discrete hazard for the dropout time,

(9) $$\begin{array}{ccc} \lambda_{i,j-1} & = & P(S_{i}=j-1|S_{i}\geq j-1,x_{S,i,j-1},b_{i1},b_{i2})\\ & = & 1-\Phi (x_{S,i,j-1}^{T}\alpha +\gamma_{1}b_{i1}+\gamma_{2}b_{i2}), \end{array}$$

where j−1=1,…,M−1, the covariate vector $x_{S,i,j-1}$ includes indicators of baseline HIV viral load groups, HIV symptomatology at baseline, indicator of ART at baseline, ${\left(j-1\right)}^{*}=(j-2)/11$ and ${\left\{{\left(j-1\right)}^{*}\right\}}^{2}$ (to account for the change in the discrete‐time hazards over time), and the interaction between ART and time ${\left(j-1\right)}^{*}$. The specification of the functional forms of the random effects in (9) is based on the belief that patients who had higher CD4 count levels at baseline (i.e., intercept) and/or who showed lower decreasing rates in their longitudinal CD4 count profiles (i.e., time slopes) are less likely to drop out.

### 3.2 Priors and posterior inference

Independent normal priors N(0,100) are assigned to β and the parameter δ in ${𝚺}_{b}$. For parameters in (9), we assign weakly informative N(0,4) priors to α, $\gamma_{1}$ and $\gamma_{2}$. For variance component parameters, we assign the prior $\sigma_{\varepsilon }^{2}∼\mathrm{Inverse}-\mathrm{Gamma}(0.001,0.001)$ and $\sigma_{k}∼\mathrm{Uniform}(0,5)$ (k=1,2) for ${𝚺}_{b}$. We run three MCMC chains with diverse initial values using the WinBUGS package 2003 and assess convergence within a 5000‐iteration burn‐in period using trace plots and Gelman and Rubin convergence statistics. The computation time is about 3.5 h on a Windows server with 2.60 GHz CPU (4 processors) and 128 GB memory when parallelizing the chains, which can be reduced if using MultiBUGS (Goudie et al., 2017), the newly released parallelized version of WinBUGS. After convergence, pooled posterior samples of size 9000 (after thinning by 5) are used for model inference.

### 3.3 Model assessment

To assess the fit of the SPM to the observed data, we use posterior predictive checks, specifically the $\chi^{2}$ discrepancy statistics described in Gelman et al. (1996) with replicated observed data, as recommended in Daniels et al. (2012) and Xu et al. (2016). Detailed steps can be found in supporting information. The posterior probability that the $\chi^{2}$ statistic is larger than the observed $\chi^{2}$ statistic is 0.212, which does not indicate lack of fit of our SPM to the observed HERS data.

### 3.4 Posterior inference

The posterior summaries for the parameters in the SPM are presented in Table 1. For comparison, we also fit a linear mixed model (LMM) that has the same form as in (8) but assumes ignorability of the dropout time and a PMM that was described in Hogan et al. (2004). Details for the PMM can be found in supporting information.

**Table 1:** Posterior mean and 95% credible intervals of the model parameters in the SPM and the LMM fitted to the HERS data

	**SPM**						**LMM**		
	**Longitudinal**			**Dropout**			**Longitudinal**		
	**Mean**	**2.5%**	**97.5%**	**Mean**	**2.5%**	**97.5%**	**Mean**	**2.5%**	**97.5%**
Intercept	−0.55	−0.75	−0.36	1.11	0.91	1.32	−0.57	−0.75	−0.38
Baseline HIV viral load									
0–500	1.52	1.32	1.74	0.75	0.54	0.97	1.54	1.33	1.74
500–5k	1.02	0.82	1.22	0.63	0.44	0.83	1.03	0.83	1.21
5k–30k	0.47	0.26	0.70	0.26	0.05	0.47	0.48	0.26	0.69
30k+ (reference)									
Baseline HIV symptoms	−0.02	−0.07	0.03	−0.01	−0.06	0.05	−0.03	−0.08	0.03
ART at baseline	−0.65	−0.77	−0.53	−0.22	−0.40	−0.04	−0.66	−0.77	−0.55
${\left(j-1\right)}^{*}$	–	–	–	1.67	1.09	2.28	–	–	–
${\left\{{\left(j-1\right)}^{*}\right\}}^{2}$	–	–	–	−2.79	−3.41	−2.16	–	–	–
${\left(j-1\right)}^{*}$*ART at baseline	–	–	–	0.37	0.04	0.70	–	–	–
Time (visit)	−1.21	−1.59	−0.84	–	–	–	−0.91	−1.29	−0.54
Time*baseline viral load									
0–500	0.59	0.21	1.00	–	–	–	0.37	−0.03	0.78
500–5k	0.53	0.15	0.91	–	–	–	0.35	−0.03	0.74
5k–30k	0.37	−0.06	0.79	–	–	–	0.25	−0.16	0.67
30k+ (reference)				–	–	–			
Time*baseline HIV symptoms	−0.06	−0.15	0.04	–	–	–	−0.04	−0.14	0.05
Time*ART at baseline	0.21	0.01	0.40	–	–	–	0.25	0.06	0.43
$\mathrm{corr}(b_{i1},b_{i2})$	−0.20	−0.29	−0.13	–	–	–	−0.23	−0.31	−0.14
$\mathrm{var}(b_{i1})$	0.56	0.50	0.62	–	–	–	0.56	0.50	0.62
$\mathrm{var}(b_{i2})$	1.24	1.07	1.44	–	–	–	1.12	0.97	1.29
$\sigma_{\varepsilon }^{2}$	0.15	0.14	0.16	–	–	–	0.15	0.14	0.16
$\gamma_{1}$	–	–	–	0.23	0.15	0.30	–	–	–
$\gamma_{2}$	–	–	–	0.28	0.22	0.35	–	–	–

The estimated main effect of time (posterior mean) from the SPM is −1.21 (95% credible interval (CI) =[−1.59, −0.84]), which is larger in magnitude than the estimate from the LMM under the ignorability assumption. The primary difference between the LMM and SPM analyses is that the LMM assumes that those who dropped out from the study had similar longitudinal CD4 profiles (intercept and time slopes) as those that did not, given past observed longitudinal data and covariates. However, from Table 1 it is clear that patients who dropped out early tended to have larger declines in CD4 count over time ($\gamma_{2}=0.28$ (95% CI =[0.22, 0.35])). As a result, the time slope under ignorability may be underestimated (with less steep decline). Similarly, the SPM estimates show larger differences in the slope of CD4 count within baseline viral load groups, while results for the dropout model in Table 1 indicate that the hazard of dropout is higher for those with higher baseline HIV viral load. Nevertheless, due to the unverifiable assumption on the extrapolation distribution in the SPM, it is essential to conduct sensitivity analysis to check the impact on the final inference for the covariate effects in the HERS population.

### 3.5 Sensitivity analysis

For sensitivity analysis, we use the specification for $\Delta_{i}$ as in (7) and assume that the sensitivity parameter *a* follows a triangular distribution with the range [−2,0] and the mode at −1. Because we standardized the visit number *j* in (8), the missing outcome $Y_{\mathrm{ik}}$ (k=j,…,12) after dropout at visit j−1 has the following form, $Y_{\mathrm{ik}}=x_{\mathrm{ik}}^{T}\beta +b_{i1}+b_{i2}k^{*}+\Delta_{i}{\left\{k^{*}-{\left(j-1\right)}^{*}\right\}}_{+}+\varepsilon_{\mathrm{ik}}$, where * stands for standardization by taking (x−1)/11. Sampling from this distribution then follows the procedure as described in Section 2.5.

To summarize the covariate effects, we use the G‐computation procedure described in Section 2.5. For presentation purpose, we fix the value of baseline HIV symptoms at zero and focus on the effects of baseline HIV viral load and ART treatment groups.

Sampling from the extrapolation distribution in the sensitivity analysis involves evaluating the posterior standard deviation of the random slope given the observed data, $\sigma_{b_{i2}}$, for each G‐computation sample. In supporting information, it can be seen that these evaluations require numerous calculations of multivariate normal probabilities, which slow down the overall G‐computation when the dimension of the multivariate normal is high (up to 11 in the HERS example). To speed up the G‐computation for the HERS analysis, we approximate $\sigma_{b_{i2}}$ using the average estimated posterior standard deviations of the random slopes for all HERS patients within each of the 8 covariate groups defined by the baseline viral load level, ART status and HIV symptoms. More details about the approximation of $\sigma_{b_{i2}}$ can be found in supporting information. We use n=82,700 Monte Carlo samples for each covariate group given a set of posterior samples of model parameters. The G‐computation is parallelized for 320 sets of posterior samples of the model parameters using the ‘parallel’ package in R on high performance clusters. It takes less than 2 h to finish the G‐computation for a set of posterior samples. This can be further reduced if the Monte Carlo samples for each posterior sample are divided into blocks for parallelization.

**Figure 2:**
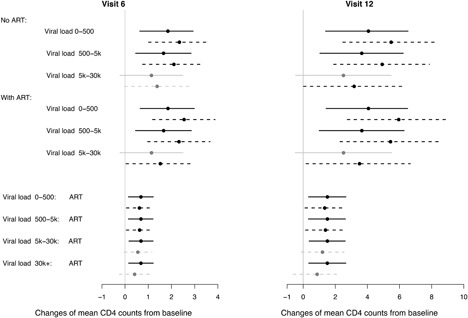
Results (posterior means and 95% credible intervals) for marginal covariate effects on changes of mean square root CD4 counts from baseline to visits 6 and 12 in the HERS analysis. Top: baseline viral load effects on mean CD4 count changes, given baseline ART status. Bottom: baseline ART status effects on mean CD4 count changes, given baseline viral load levels. Solid lines (—–): 95% credible intervals under the default extrapolation distribution of the SPM; dashed lines (‐ ‐ ‐ ‐ ‐): 95% credible intervals under the extrapolation distribution in the sensitivity analysis. The estimated effects with 95% credible intervals covering zero and not covering zero are in gray and black, respectively.

Note that the marginal covariate effects in the sensitivity analysis no longer follow a linear form as in the fitted SPM, i.e., there are interactions between covariates. Therefore we provide the effects of baseline viral load level given the ART status, and also the effects of ART status given the baseline viral load level, on the changes of mean CD4 counts from baseline to visits 6 and 12 in Figure 2. The top of Figure 2 shows the differences of the mean CD4 count changes between three baseline viral load groups and the reference group (>30,000), given the ART status. The estimated viral load effects in the sensitivity analysis are all larger than those in the SPM. This is because the mean CD4 counts are adjusted downwards in the sensitivity analysis compared with the SPM estimates, and the adjustment is biggest for the group with highest viral load (reference group) which was more likely to drop out. As a result, conclusions about the viral load effects differ in the two analyses. For example, in both analyses the viral load (5000,30,000] group is associated with smaller decreases in mean CD4 counts from baseline to visits 6 and 12, compared with the highest viral load group. But in the sensitivity analysis, the 95% CIs for these effects no longer cover zero, unlike in the SPM. Similarly, conclusions about the effects of the ART status also differ between the two analyses. For example, the effects of the ART status (the bottom of Figure 2) have been reduced in the sensitivity analysis, in particular, the 95% CIs for the ART effects in the higher viral load groups ((5000,30,000], >30,000), now cover zero.

Overall, despite these differences, it appears that the conclusions of the covariate effects from the SPM are not overly sensitive to the deviations considered here. For example, given other baseline covariates, patients with higher baseline viral load had larger decreases of mean CD4 counts compared with patients with lower baseline viral load. This is also consistent with the findings from the PMM; see details in supporting information.

## 4 Conclusion and discussion

In this article we proposed a new sensitivity analysis approach for informative dropout using SPMs. The distinctive feature of our approach is that the inference for observed data is not influenced by the global sensitivity parameter, which follows the principle as proposed by Daniels and Hogan (2008) in a full probability model based setting. We showed that the default extrapolation distribution under the SPM specified here is available in a closed form. Therefore it is convenient to anchor our sensitivity analysis at this default extrapolation distribution. In addition, using the HERS data, we demonstrated that the deviation of the extrapolation distribution specified in the sensitivity analysis from the default can be calibrated using the observed data for each patient who dropped out.

Sensitivity analysis approaches for informative dropout based on selection models and PMMs have also been proposed in the literature. In selection models, the sensitivity parameter is specified in the selection function (e.g., the regression coefficients in the dropout model). However, with *parametric* models for the longitudinal outcome and the selection function, altering the sensitivity parameter in the selection function will also affect the model fit to the observed data, which is not consistent with the principle of sensitivity analyses 2008. Since SPMs are also parametric, we anchor our sensitivity analysis at the extrapolation distribution of the missing outcomes, not at the selection function, similarly to the sensitivity analysis approach based on PMMs. We provide a more detailed discussion on sensitivity analysis based on PMMs in supporting information.

Because we specified a piece‐wise linear model for the individual longitudinal profile and the random intercept $b_{i1}$ reflects the CD4 count level at baseline of the HERS where data are complete, we did not connect $b_{i1}$ to the sensitivity parameter. However, if we follow the approach in Linero and Daniels (2015), we can specify the sensitivity parameter to represent a location shift from $b_{i1}+b_{i2}t$, where *t* is a time point after dropout. This location‐shift model can also be used in a SPM with informative intermittent missing data, where the series of missing data indicators are modeled using a probit model. It is straightforward to show that the default extrapolation distribution under this SPM is also skew‐normal that depends on *all observed* outcome data (not only the observed outcome data up to the current visit with the intermittent missing data), covariates, and model parameters. The sensitivity analysis can again be anchored at this default extrapolation distribution and we then specify a location shift model for the deviation from the default extrapolation distribution that is again controlled by a global sensitivity parameter. The final inference under the SPM and sensitivity analysis can be provided through G‐computation.

Using a probit model for the discrete hazard of dropout, the SPM used in our approach benefits from a closed form of the default extrapolation distribution. The probit link used in the dropout model not only facilitates sensitivity analysis, but also naturally reflects the assumption that the discrete hazard of dropout depends on the *normally* distributed random effects that characterize underlying individual longitudinal profiles. Other models, e.g., logistic models, can also be used in a SPM. However, in such models, the default extrapolation distributions are not available in closed forms. To approximate them, we can first sample the posterior distribution of the random effects using the Metropolis–Hastings algorithm and then sample the missing outcomes using the longitudinal model specified in the SPM and the samples of random effects and other model parameters. This is similar to the algorithm used for dynamic predictions based on SPMs described in Rizopoulos (2011).

The general approach for sensitivity analysis proposed here is similar in spirit to the framework proposed by Linero and Daniels (2015) and Linero (2017), where a flexible ‘working model’ for the joint distribution of the complete longitudinal outcomes and the dropout time is specified and identifying restrictions are then applied when performing sensitivity analyses with the extrapolation distribution. The typical SPM can be thought of as the ‘working model’ described in these articles. Here, however, we recommend performing sensitivity analysis grounded off the extrapolation distribution from the ‘working model’, unlike anchoring at the MAR restrictions as done in Linero and Daniels (2015) and Linero (2017).

## Supporting information

Additional supporting information may be found online in the Supporting Information section at the end of the article.

Supplementary Materials.
