# Supplementary material for: A sensitivity analysis approach for informative dropout using shared parameter models
Source: Biometrics. 2019 Apr 1;75(3):917–26. doi: 10.1111/biom.13027 (PMC6739227; doi:10.1111/biom.13027)
Supplement: Supplementary file 1 — Supplementary Materials. [file BIOM-75-917-s001.zip › SA_SPM_supplementary_r2.pdf]

Biometrics 000, 000–000 DOI: 000
000 0000

Supporting Information for 'A Sensitivity Analysis Approach for Informative Dropout using Shared Parameter Models'

by Li Su, Qiuju Li, Jessica K. Barrett and Michael J. Daniels

This paper has been submitted for consideration for publication in *Biometrics*

1. Preliminaries

In this section we describe some preliminary material for the proofs presented in Sections 2, 3 and 5.

1.1 *Multivariate skew-normal distribution*

Let

$$\mathbf{y} = \boldsymbol{\mu} + \mathbf{E}_1,$$

$$\mathbf{z} = -\boldsymbol{\nu} + \mathbf{D}\mathbf{E}_1 + \mathbf{E}_2,$$

where $\mathbf{E}_1 \sim N^{(q)}(\mathbf{0}, \boldsymbol{\Sigma})$ and $\mathbf{E}_2 \sim N^{(p)}(\mathbf{0}, \boldsymbol{\Delta})$ are independent random vectors, $\boldsymbol{\mu}$ is a $q \times 1$ vector, $\boldsymbol{\nu}$ is a $p \times 1$ vector, and $\mathbf{D}_{p\times q}$ is a matrix. The joint distribution of $\mathbf{y}$ and $\mathbf{z}$ is

$$\begin{pmatrix} \mathbf{y} \\ \mathbf{z} \end{pmatrix} \sim N^{(q+p)} \left[\begin{pmatrix} \boldsymbol{\mu} \\ -\boldsymbol{\nu} \end{pmatrix}, \begin{pmatrix} \boldsymbol{\Sigma} & \boldsymbol{\Sigma}\mathbf{D}^{\mathrm{T}} \\ \mathbf{D}\boldsymbol{\Sigma} & \boldsymbol{\Delta} + \mathbf{D}\boldsymbol{\Sigma}\mathbf{D}^{\mathrm{T}} \end{pmatrix} \right].$$

Since

$$f_{\mathbf{y}|\{\mathbf{z}\geqslant\mathbf{0}\}}(\mathbf{y}|\mathbf{z} \geqslant \mathbf{0}) = \frac{f_{\mathbf{y}}(\mathbf{y})}{P(\mathbf{z} \geqslant \mathbf{0})} P(\mathbf{z} \geqslant \mathbf{0}|\mathbf{y} = \mathbf{y}),$$

the conditional density of $\mathbf{y}$ given $\mathbf{z} \geqslant \mathbf{0}$ has the following form,

$$f(\mathbf{y}; \boldsymbol{\mu}, \boldsymbol{\Sigma}, \mathbf{D}, \boldsymbol{\nu}, \boldsymbol{\Delta}) = \phi^{(q)}(\mathbf{y}; \boldsymbol{\mu}, \boldsymbol{\Sigma}) \frac{\Phi^{(p)}(\mathbf{D}(\mathbf{y} - \boldsymbol{\mu}); \boldsymbol{\nu}, \boldsymbol{\Delta})}{\Phi^{(p)}(\mathbf{0}; \boldsymbol{\nu}, \boldsymbol{\Delta} + \mathbf{D}\boldsymbol{\Sigma}\mathbf{D}^{\mathrm{T}})}, \tag{1}$$

where $\phi^{(q)}(\cdot; \boldsymbol{\eta}, \boldsymbol{\Psi})$ and $\Phi^{(q)}(\cdot; \boldsymbol{\eta}, \boldsymbol{\Psi})$ are the density and distribution function of a q-dimensional normal distribution with mean $\boldsymbol{\eta}$ and covariance matrix $\boldsymbol{\Psi}$. The density function in (1) is from the class of multivariate skew-normal distribution described in González-Farías et al. (2004), Arnold (2009) and Flecher et al. (2009). Following González-Farías et al. (2004), we let $CSN_{q,p}(\boldsymbol{\mu}, \boldsymbol{\Sigma}, \mathbf{D}, \boldsymbol{\nu}, \boldsymbol{\Delta})$ denote this multivariate skew-normal distribution. Its moment generating function is

$$\mathbf{M}_Y(\mathbf{t}) = \exp\left\{ \mathbf{t}^{\mathrm{T}}\boldsymbol{\mu} + \frac{1}{2}\mathbf{t}^{\mathrm{T}}\boldsymbol{\Sigma}\mathbf{t} \right\} \frac{\Phi^{(p)}(\mathbf{D}\boldsymbol{\Sigma}\mathbf{t}; \boldsymbol{\nu}, \boldsymbol{\Delta} + \mathbf{D}\boldsymbol{\Sigma}\mathbf{D}^{\mathrm{T}})}{\Phi^{(p)}(\mathbf{0}; \boldsymbol{\nu}, \boldsymbol{\Delta} + \mathbf{D}\boldsymbol{\Sigma}\mathbf{D}^{\mathrm{T}})}.$$

The multivariate normal distribution is a special case of the multivariate skew-normal distribution when $\mathbf{D} = \mathbf{0}$.

Sampling from $CSN_{q,p}(\boldsymbol{\mu}, \boldsymbol{\Sigma}, \mathbf{D}, \boldsymbol{\nu}, \boldsymbol{\Delta})$ can proceed by drawing from $\mathbf{z} \sim N^{(p)}(-\boldsymbol{\nu}, \boldsymbol{\Delta} + \mathbf{D}\boldsymbol{\Sigma}\mathbf{D}^{\mathrm{T}})$ until $\mathbf{z} \geqslant \mathbf{0}$, then draw $\mathbf{y}$ from $N^{(q)}\left\{\boldsymbol{\mu} + (\mathbf{D}\boldsymbol{\Sigma})^{\mathrm{T}}(\boldsymbol{\Delta} + \mathbf{D}\boldsymbol{\Sigma}\mathbf{D}^{\mathrm{T}})^{-1}(\mathbf{z}+\boldsymbol{\nu}), \boldsymbol{\Sigma} - (\mathbf{D}\boldsymbol{\Sigma})^{\mathrm{T}}(\boldsymbol{\Delta} + \mathbf{D}\boldsymbol{\Sigma}\mathbf{D}^{\mathrm{T}})^{-1}(\mathbf{D}\boldsymbol{\Sigma})\right\}$.

1.1.1 *Mean.* Note that

$$\begin{aligned}\frac{\partial \mathbf{M}_Y(\mathbf{t})}{\partial \mathbf{t}} =& \exp\left\{\mathbf{t}^{\mathrm{T}}\boldsymbol{\mu} + \frac{1}{2}\mathbf{t}^{\mathrm{T}}\boldsymbol{\Sigma}\mathbf{t}\right\}\frac{\Phi^{(p)}(\mathbf{D}\boldsymbol{\Sigma}\mathbf{t}; \boldsymbol{\nu}, \boldsymbol{\Delta} + \mathbf{D}\boldsymbol{\Sigma}\mathbf{D}^{\mathrm{T}})}{\Phi^{(p)}(\mathbf{0}; \boldsymbol{\nu}, \boldsymbol{\Delta} + \mathbf{D}\boldsymbol{\Sigma}\mathbf{D}^{\mathrm{T}})}(\boldsymbol{\mu} + \boldsymbol{\Sigma}\mathbf{t}) \\ &+ \exp\left\{\mathbf{t}^{\mathrm{T}}\boldsymbol{\mu} + \frac{1}{2}\mathbf{t}^{\mathrm{T}}\boldsymbol{\Sigma}\mathbf{t}\right\}\frac{\partial\Phi^{(p)}(\mathbf{D}\boldsymbol{\Sigma}\mathbf{t}; \boldsymbol{\nu}, \boldsymbol{\Delta} + \mathbf{D}\boldsymbol{\Sigma}\mathbf{D}^{\mathrm{T}})/\partial\mathbf{t}}{\Phi^{(p)}(\mathbf{0}; \boldsymbol{\nu}, \boldsymbol{\Delta} + \mathbf{D}\boldsymbol{\Sigma}\mathbf{D}^{\mathrm{T}})}.\end{aligned}$$

So the mean of the multivariate skew-normal distribution is

$$\begin{aligned}\mathrm{E}(\mathbf{y}; \boldsymbol{\mu}, \boldsymbol{\Sigma}, \mathbf{D}, \boldsymbol{\nu}, \boldsymbol{\Delta}) =& \frac{\partial \mathbf{M}_Y(\mathbf{t})}{\partial \mathbf{t}}|_{\mathbf{t}=0} \\ =& \boldsymbol{\mu} + \frac{\partial\Phi^{(p)}(\mathbf{D}\boldsymbol{\Sigma}\mathbf{t}; \boldsymbol{\nu}, \boldsymbol{\Delta} + \mathbf{D}\boldsymbol{\Sigma}\mathbf{D}^{\mathrm{T}})/\partial\mathbf{t}|_{\mathbf{t}=0}}{\Phi^{(p)}(\mathbf{0}; \boldsymbol{\nu}, \boldsymbol{\Delta} + \mathbf{D}\boldsymbol{\Sigma}\mathbf{D}^{\mathrm{T}})}.\end{aligned}$$

For details about obtaining the derivatives of a multivariate normal distribution function with respect to $\mathbf{t}$, see Section A.2.

1.1.2 *Variance.* Note that

$$\begin{aligned}\frac{\partial^2 \mathbf{M}_Y(\mathbf{t})}{\partial \mathbf{t}\partial\mathbf{t}^{\mathrm{T}}} =& \exp\left\{\mathbf{t}^{\mathrm{T}}\boldsymbol{\mu} + \frac{1}{2}\mathbf{t}^{\mathrm{T}}\boldsymbol{\Sigma}\mathbf{t}\right\}\frac{\Phi^{(p)}(\mathbf{D}\boldsymbol{\Sigma}\mathbf{t}; \boldsymbol{\nu}, \boldsymbol{\Delta} + \mathbf{D}\boldsymbol{\Sigma}\mathbf{D}^{\mathrm{T}})}{\Phi^{(p)}(\mathbf{0}; \boldsymbol{\nu}, \boldsymbol{\Delta} + \mathbf{D}\boldsymbol{\Sigma}\mathbf{D}^{\mathrm{T}})}(\boldsymbol{\mu} + \boldsymbol{\Sigma}\mathbf{t})(\boldsymbol{\mu} + \boldsymbol{\Sigma}\mathbf{t})^{\mathrm{T}} \\ &+ \exp\left\{\mathbf{t}^{\mathrm{T}}\boldsymbol{\mu} + \frac{1}{2}\mathbf{t}^{\mathrm{T}}\boldsymbol{\Sigma}\mathbf{t}\right\}(\boldsymbol{\mu} + \boldsymbol{\Sigma}\mathbf{t})\frac{\left(\partial\Phi^{(p)}(\mathbf{D}\boldsymbol{\Sigma}\mathbf{t}; \boldsymbol{\nu}, \boldsymbol{\Delta} + \mathbf{D}\boldsymbol{\Sigma}\mathbf{D}^{\mathrm{T}})/\partial\mathbf{t}\right)^{\mathrm{T}}}{\Phi^{(p)}(\mathbf{0}; \boldsymbol{\nu}, \boldsymbol{\Delta} + \mathbf{D}\boldsymbol{\Sigma}\mathbf{D}^{\mathrm{T}})} \\ &+ \exp\left\{\mathbf{t}^{\mathrm{T}}\boldsymbol{\mu} + \frac{1}{2}\mathbf{t}^{\mathrm{T}}\boldsymbol{\Sigma}\mathbf{t}\right\}\frac{\Phi^{(p)}(\mathbf{D}\boldsymbol{\Sigma}\mathbf{t}; \boldsymbol{\nu}, \boldsymbol{\Delta} + \mathbf{D}\boldsymbol{\Sigma}\mathbf{D}^{\mathrm{T}})}{\Phi^{(p)}(\mathbf{0}; \boldsymbol{\nu}, \boldsymbol{\Delta} + \mathbf{D}\boldsymbol{\Sigma}\mathbf{D}^{\mathrm{T}})}\boldsymbol{\Sigma} \\ &+ \exp\left\{\mathbf{t}^{\mathrm{T}}\boldsymbol{\mu} + \frac{1}{2}\mathbf{t}^{\mathrm{T}}\boldsymbol{\Sigma}\mathbf{t}\right\}\frac{\partial\Phi^{(p)}(\mathbf{D}\boldsymbol{\Sigma}\mathbf{t}; \boldsymbol{\nu}, \boldsymbol{\Delta} + \mathbf{D}\boldsymbol{\Sigma}\mathbf{D}^{\mathrm{T}})/\partial\mathbf{t}}{\Phi^{(p)}(\mathbf{0}; \boldsymbol{\nu}, \boldsymbol{\Delta} + \mathbf{D}\boldsymbol{\Sigma}\mathbf{D}^{\mathrm{T}})}(\boldsymbol{\mu} + \boldsymbol{\Sigma}\mathbf{t})^{\mathrm{T}} \\ &+ \exp\left\{\mathbf{t}^{\mathrm{T}}\boldsymbol{\mu} + \frac{1}{2}\mathbf{t}^{\mathrm{T}}\boldsymbol{\Sigma}\mathbf{t}\right\}\frac{\partial^2\Phi^{(p)}(\mathbf{D}\boldsymbol{\Sigma}\mathbf{t}; \boldsymbol{\nu}, \boldsymbol{\Delta} + \mathbf{D}\boldsymbol{\Sigma}\mathbf{D}^{\mathrm{T}})/\partial\mathbf{t}\partial\mathbf{t}^{\mathrm{T}}}{\Phi^{(p)}(\mathbf{0}; \boldsymbol{\nu}, \boldsymbol{\Delta} + \mathbf{D}\boldsymbol{\Sigma}\mathbf{D}^{\mathrm{T}})}.\end{aligned}$$

Thus the 2nd moment of the multivariate skew-normal distribution is

$$\begin{aligned}\frac{\partial^2 \mathbf{M}_Y(\mathbf{t})}{\partial \mathbf{t}\partial\mathbf{t}^{\mathrm{T}}}|_{\mathbf{t}=0} =& \boldsymbol{\mu}\boldsymbol{\mu}^{\mathrm{T}} + \boldsymbol{\Sigma} + \boldsymbol{\mu}\frac{\left(\partial\Phi^{(p)}(\mathbf{D}\boldsymbol{\Sigma}\mathbf{t}; \boldsymbol{\nu}, \boldsymbol{\Delta} + \mathbf{D}\boldsymbol{\Sigma}\mathbf{D}^{\mathrm{T}})/\partial\mathbf{t}|_{\mathbf{t}=0}\right)^{\mathrm{T}}}{\Phi^{(p)}(\mathbf{0}; \boldsymbol{\nu}, \boldsymbol{\Delta} + \mathbf{D}\boldsymbol{\Sigma}\mathbf{D}^{\mathrm{T}})} \\ &+ \frac{\partial\Phi^{(p)}(\mathbf{D}\boldsymbol{\Sigma}\mathbf{t}; \boldsymbol{\nu}, \boldsymbol{\Delta} + \mathbf{D}\boldsymbol{\Sigma}\mathbf{D}^{\mathrm{T}})/\partial\mathbf{t}|_{\mathbf{t}=0}}{\Phi^{(p)}(\mathbf{0}; \boldsymbol{\nu}, \boldsymbol{\Delta} + \mathbf{D}\boldsymbol{\Sigma}\mathbf{D}^{\mathrm{T}})}\boldsymbol{\mu}^{\mathrm{T}} \\ &+ \frac{\partial^2\Phi^{(p)}(\mathbf{D}\boldsymbol{\Sigma}\mathbf{t}; \boldsymbol{\nu}, \boldsymbol{\Delta} + \mathbf{D}\boldsymbol{\Sigma}\mathbf{D}^{\mathrm{T}})/\partial\mathbf{t}\partial\mathbf{t}^{\mathrm{T}}|_{\mathbf{t}=0}}{\Phi^{(p)}(\mathbf{0}; \boldsymbol{\nu}, \boldsymbol{\Delta} + \mathbf{D}\boldsymbol{\Sigma}\mathbf{D}^{\mathrm{T}})},\end{aligned}$$

and the variance of the multivariate skew-normal distribution is

$$\begin{aligned}\mathrm{Var}(\mathbf{y};\boldsymbol{\mu},\boldsymbol{\Sigma},\mathbf{D},\boldsymbol{\nu},\boldsymbol{\Delta}) =&\frac{\partial^2\mathbf{M}_Y(\mathbf{t})}{\partial\mathbf{t}\partial\mathbf{t}^{\mathrm{T}}}|_{\mathbf{t}=0} - \frac{\partial\mathbf{M}_Y(\mathbf{t})}{\partial\mathbf{t}}|_{\mathbf{t}=0}\left(\frac{\partial\mathbf{M}_Y(\mathbf{t})}{\partial\mathbf{t}}|_{\mathbf{t}=0}\right)^{\mathrm{T}}\\ =&\boldsymbol{\Sigma} + \frac{\partial^2\Phi^{(p)}(\mathbf{D}\boldsymbol{\Sigma}\mathbf{t};\boldsymbol{\nu},\boldsymbol{\Delta}+\mathbf{D}\boldsymbol{\Sigma}\mathbf{D}^{\mathrm{T}})/\partial\mathbf{t}\partial\mathbf{t}^{\mathrm{T}}|_{\mathbf{t}=0}}{\Phi^{(p)}(\mathbf{0};\boldsymbol{\nu},\boldsymbol{\Delta}+\mathbf{D}\boldsymbol{\Sigma}\mathbf{D}^{\mathrm{T}})}\\ &-\frac{\partial\Phi^{(p)}(\mathbf{D}\boldsymbol{\Sigma}\mathbf{t};\boldsymbol{\nu},\boldsymbol{\Delta}+\mathbf{D}\boldsymbol{\Sigma}\mathbf{D}^{\mathrm{T}})/\partial\mathbf{t}|_{\mathbf{t}=0}}{\Phi^{(p)}(\mathbf{0};\boldsymbol{\nu},\boldsymbol{\Delta}+\mathbf{D}\boldsymbol{\Sigma}\mathbf{D}^{\mathrm{T}})}\\ &\left(\frac{\partial\Phi^{(p)}(\mathbf{D}\boldsymbol{\Sigma}\mathbf{t};\boldsymbol{\nu},\boldsymbol{\Delta}+\mathbf{D}\boldsymbol{\Sigma}\mathbf{D}^{\mathrm{T}})/\partial\mathbf{t}|_{\mathbf{t}=0}}{\Phi^{(p)}(\mathbf{0};\boldsymbol{\nu},\boldsymbol{\Delta}+\mathbf{D}\boldsymbol{\Sigma}\mathbf{D}^{\mathrm{T}})}\right)^{\mathrm{T}}.\end{aligned}$$

1.2 *Derivatives of a multivariate normal distribution function*

Let $\Phi^{(p)}(\mathbf{G}\mathbf{t};\boldsymbol{\nu},\boldsymbol{\Psi})$ denote the cumulative distribution function (cdf) of a p-dimensional multivariate normal distribution with mean $\boldsymbol{\nu}$ and covariance matrix $\boldsymbol{\Psi}$, where $\mathbf{G}$ is a $p\times q$ matrix, $\mathbf{t}$ is a $q\times 1$ vector, $\boldsymbol{\nu}$ is a $p\times 1$ vector and $\boldsymbol{\Psi}$ is a $p\times p$ covariance matrix. The first derivative of $\Phi^{(p)}(\mathbf{G}\mathbf{t};\boldsymbol{\nu},\boldsymbol{\Psi})$ with respect to $\mathbf{t}$ is

$$\frac{\partial\Phi^{(p)}(\mathbf{G}\mathbf{t};\boldsymbol{\nu},\boldsymbol{\Psi})}{\partial\mathbf{t}} = \frac{\partial}{\partial\mathbf{t}}\int_{-\infty}^{\mathbf{G}\mathbf{t}}\phi^{(p)}(\mathbf{z};\boldsymbol{\nu},\boldsymbol{\Psi})dz_1dz_2\cdots dz_p$$

$$=\frac{\partial}{\partial\mathbf{t}}\int_{-\infty}^{\mathbf{G}_{1\cdot}\mathbf{t}}\int_{-\infty}^{\mathbf{G}_{2\cdot}\mathbf{t}}\cdots\int_{-\infty}^{\mathbf{G}_{p\cdot}\mathbf{t}}\phi^{(p)}(\mathbf{z};\boldsymbol{\nu},\boldsymbol{\Psi})dz_1dz_2\cdots dz_p$$

$$=\frac{\partial}{\partial\mathbf{t}}\int_{-\infty}^{\mathbf{G}_{\ell\cdot}\mathbf{t}}\left[\int_{-\infty}^{\mathbf{G}_{-\ell\cdot}\mathbf{t}}\phi^{(p-1)}\left\{\mathbf{z}_{-\ell};\boldsymbol{\nu}_{-\ell}+\boldsymbol{\Psi}_{-\ell,\ell}\boldsymbol{\Psi}_{\ell,\ell}^{-1}(\mathbf{z}_\ell-\boldsymbol{\nu}_\ell),\boldsymbol{\Psi}_{-\ell,-\ell}-\boldsymbol{\Psi}_{-\ell,\ell}\boldsymbol{\Psi}_{\ell,\ell}^{-1}\boldsymbol{\Psi}_{\ell,-\ell}\right\}d\mathbf{z}_{-\ell}\right]$$

$$\phi^{(1)}(\mathbf{z}_\ell;\boldsymbol{\nu}_\ell,\boldsymbol{\Psi}_{\ell,\ell})d\mathbf{z}_\ell$$

$$=\sum_{\ell=1}^{p}\Phi^{(p-1)}\left\{\mathbf{G}_{-\ell\cdot}\mathbf{t};\boldsymbol{\nu}_{-\ell}+\boldsymbol{\Psi}_{-\ell,\ell}\boldsymbol{\Psi}_{\ell,\ell}^{-1}(\mathbf{G}_{\ell\cdot}\mathbf{t}-\boldsymbol{\nu}_\ell),\boldsymbol{\Psi}_{-\ell,-\ell}-\boldsymbol{\Psi}_{-\ell,\ell}\boldsymbol{\Psi}_{\ell,\ell}^{-1}\boldsymbol{\Psi}_{\ell,-\ell}\right\}$$

$$\phi^{(1)}(\mathbf{G}_{\ell\cdot}\mathbf{t};\boldsymbol{\nu}_\ell,\boldsymbol{\Psi}_{\ell,\ell})\mathbf{G}_{\ell\cdot}^{\mathrm{T}}$$

$$=\sum_{\ell=1}^{p}\Phi^{(p-1)}\left\{\left(\mathbf{G}_{-\ell\cdot}-\boldsymbol{\Psi}_{-\ell,\ell}\boldsymbol{\Psi}_{\ell,\ell}^{-1}\mathbf{G}_{\ell\cdot}\right)\mathbf{t};\boldsymbol{\nu}_{-\ell}-\boldsymbol{\Psi}_{-\ell,\ell}\boldsymbol{\Psi}_{\ell,\ell}^{-1}\boldsymbol{\nu}_\ell,\boldsymbol{\Psi}_{-\ell,-\ell}-\boldsymbol{\Psi}_{-\ell,\ell}\boldsymbol{\Psi}_{\ell,\ell}^{-1}\boldsymbol{\Psi}_{\ell,-\ell}\right\}$$

$$\phi^{(1)}(\mathbf{G}_{\ell\cdot}\mathbf{t};\boldsymbol{\nu}_\ell,\boldsymbol{\Psi}_{\ell,\ell})\mathbf{G}_{\ell\cdot}^{\mathrm{T}}$$

where $\mathbf{G}_{\ell\cdot}$ is the ℓth row vector of $\mathbf{G}$, $\mathbf{G}_{-\ell\cdot}$ is the matrix $\mathbf{G}$ with its ℓth row removed, $\mathbf{z}=(z_1,\ldots,z_p)^{\mathrm{T}}$, $\mathbf{z}_\ell$ is the ℓth element of vector $\mathbf{z}$, $\mathbf{z}_{-\ell}$ is the vector $\mathbf{z}$ with its ℓth element removed, $\boldsymbol{\nu}_\ell$ is the ℓth element of vector $\boldsymbol{\nu}$, $\boldsymbol{\nu}_{-\ell}$ is the vector $\boldsymbol{\nu}$ with its ℓth element removed, $\boldsymbol{\Psi}_{-\ell,k}$ is

the kth column vector of $\boldsymbol{\Psi}$ after removing its ℓth row, $\boldsymbol{\Psi}_{\ell,-k}$ is the ℓth row vector of $\boldsymbol{\Psi}$ after removing its kth column, $\boldsymbol{\Psi}_{-\ell,-k}$ is the matrix of $\boldsymbol{\Psi}$ with its ℓth row and k^{th} column both removed, and $\boldsymbol{\Psi}_{\ell,k}$ is the $(\ell,k)^{th}$ element of the matrix $\boldsymbol{\Psi}$.

Denote $\mathbf{G}^{\ell *} = \mathbf{G}_{-\ell\cdot} - \boldsymbol{\Psi}_{-\ell,\ell}\boldsymbol{\Psi}_{\ell,\ell}^{-1}\mathbf{G}_{\ell\cdot}$, $\boldsymbol{\nu}^{\ell *} = \boldsymbol{\nu}_{-\ell} - \boldsymbol{\Psi}_{-\ell,\ell}\boldsymbol{\Psi}_{\ell,\ell}^{-1}\boldsymbol{\nu}_{\ell}$, $\boldsymbol{\Psi}^{\ell *} = \boldsymbol{\Psi}_{-\ell,-\ell} - \boldsymbol{\Psi}_{-\ell,\ell}\boldsymbol{\Psi}_{\ell,\ell}^{-1}\boldsymbol{\Psi}_{\ell,-\ell}$. Then the second derivative of $\Phi^{(p)}(\mathbf{G}\mathbf{t};\boldsymbol{\nu},\boldsymbol{\Psi})$ with respect to $\mathbf{t}$ is

$$\frac{\partial^2\Phi^{(p)}(\mathbf{G}\mathbf{t};\boldsymbol{\nu},\boldsymbol{\Psi})}{\partial\mathbf{t}\partial\mathbf{t}^{\mathrm{T}}} = \sum_{\ell=1}^{p}\frac{\partial}{\partial\mathbf{t}^{\mathrm{T}}}\left\{\Phi^{(p-1)}\Big(\mathbf{G}^{\ell *}\mathbf{t};\boldsymbol{\nu}^{\ell *},\boldsymbol{\Psi}^{\ell *}\Big)\phi^{(1)}(\mathbf{G}_{\ell\cdot}\mathbf{t};\boldsymbol{\nu}_{\ell},\boldsymbol{\Psi}_{\ell,\ell})\mathbf{G}_{\ell\cdot}^{\mathrm{T}}\right\}$$

$$= \sum_{\ell=1}^{p}\sum_{k=1}^{p-1}\Phi^{(p-2)}\left[\left\{\mathbf{G}_{-k\cdot}^{\ell *} - \boldsymbol{\Psi}_{-k,k}^{\ell *}(\boldsymbol{\Psi}_{k,k}^{\ell *})^{-1}\mathbf{G}_{k\cdot}^{\ell *}\right\}\mathbf{t};\boldsymbol{\nu}_{-k}^{\ell *} - \boldsymbol{\Psi}_{-k,k}^{\ell *}(\boldsymbol{\Psi}_{k,k}^{\ell *})^{-1}\boldsymbol{\nu}_{k}^{\ell *},\right.$$

$$\left.\boldsymbol{\Psi}_{-k,-k}^{\ell *} - \boldsymbol{\Psi}_{-k,k}^{\ell *}(\boldsymbol{\Psi}_{k,k}^{\ell *})^{-1}\boldsymbol{\Psi}_{k,-k}^{\ell *}\right]\phi^{(1)}(\mathbf{G}_{k\cdot}^{\ell *}\mathbf{t};\boldsymbol{\nu}_{k}^{\ell *},\boldsymbol{\Psi}_{k,k}^{\ell *})\phi^{(1)}(\mathbf{G}_{\ell\cdot}\mathbf{t};\boldsymbol{\nu}_{\ell},\boldsymbol{\Psi}_{\ell,\ell})\mathbf{G}_{\ell\cdot}^{\mathrm{T}}\mathbf{G}_{k\cdot}^{\ell *}$$

$$+\sum_{\ell=1}^{p}\Phi^{(p-1)}\Big(\mathbf{G}^{\ell *}\mathbf{t};\boldsymbol{\nu}^{\ell *},\boldsymbol{\Psi}^{\ell *}\Big)\phi^{(1)}(\mathbf{G}_{\ell\cdot}\mathbf{t};\boldsymbol{\nu}_{\ell},\boldsymbol{\Psi}_{\ell,\ell})(-\boldsymbol{\Psi}_{\ell,\ell})^{-1}(\mathbf{G}_{\ell\cdot}\mathbf{t}-\boldsymbol{\nu}_{\ell})\mathbf{G}_{\ell\cdot}^{\mathrm{T}}\mathbf{G}_{\ell\cdot}$$

Setting $\mathbf{t}=\mathbf{0}$, we have

$$\frac{\partial\Phi^{(p)}(\mathbf{G}\mathbf{t};\boldsymbol{\nu},\boldsymbol{\Psi})}{\partial\mathbf{t}}\Big|_{\mathbf{t}=\mathbf{0}}$$

$$= \sum_{\ell=1}^{p}\Phi^{(p-1)}\Big(\mathbf{0};\boldsymbol{\nu}_{-\ell} - \boldsymbol{\Psi}_{-\ell,\ell}\boldsymbol{\Psi}_{\ell,\ell}^{-1}\boldsymbol{\nu}_{\ell},\boldsymbol{\Psi}_{-\ell,-\ell} - \boldsymbol{\Psi}_{-\ell,\ell}\boldsymbol{\Psi}_{\ell,\ell}^{-1}\boldsymbol{\Psi}_{\ell,-\ell}\Big)\phi^{(1)}(0;\boldsymbol{\nu}_{\ell},\boldsymbol{\Psi}_{\ell,\ell})\mathbf{G}_{\ell\cdot}^{\mathrm{T}}$$

and

$$\frac{\partial^2\Phi^{(p)}(\mathbf{G}\mathbf{t};\boldsymbol{\nu},\boldsymbol{\Psi})}{\partial\mathbf{t}\partial\mathbf{t}^{\mathrm{T}}}\Big|_{\mathbf{t}=\mathbf{0}}$$

$$= \sum_{\ell=1}^{p}\sum_{k=1}^{p-1}\left[\Phi^{(p-2)}\left\{\mathbf{0};\boldsymbol{\nu}_{-k}^{\ell *} - \boldsymbol{\Psi}_{-k,k}^{\ell *}(\boldsymbol{\Psi}_{k,k}^{\ell *})^{-1}\boldsymbol{\nu}_{k}^{\ell *},\boldsymbol{\Psi}_{-k,-k}^{\ell *} - \boldsymbol{\Psi}_{-k,k}^{\ell *}(\boldsymbol{\Psi}_{k,k}^{\ell *})^{-1}\boldsymbol{\Psi}_{k,-k}^{\ell *}\right\}\right.$$

$$\left.\times\phi^{(1)}(0;\boldsymbol{\nu}_{k}^{\ell *},\boldsymbol{\Psi}_{k,k}^{\ell *})\phi^{(1)}(0;\boldsymbol{\nu}_{\ell},\boldsymbol{\Psi}_{\ell,\ell})\mathbf{G}_{\ell\cdot}^{\mathrm{T}}\mathbf{G}_{k\cdot}^{\ell *}\right]$$

$$+\sum_{\ell=1}^{p}\Phi^{(p-1)}\Big(\mathbf{0};\boldsymbol{\nu}^{\ell *},\boldsymbol{\Psi}^{\ell *}\Big)\phi^{(1)}(0;\boldsymbol{\nu}_{\ell},\boldsymbol{\Psi}_{\ell,\ell})\boldsymbol{\Psi}_{\ell,\ell}^{-1}\boldsymbol{\nu}_{\ell}\mathbf{G}_{\ell\cdot}^{\mathrm{T}}\mathbf{G}_{\ell\cdot}.$$

In particular, when $p=1$ we have

$$\frac{\partial\Phi^{(1)}(\mathbf{G}\mathbf{t};\boldsymbol{\nu},\boldsymbol{\Psi})}{\partial\mathbf{t}}\Big|_{\mathbf{t}=\mathbf{0}} = \phi^{(1)}(0;\boldsymbol{\nu},\boldsymbol{\Psi})\mathbf{G}^{\mathrm{T}},$$

$$\frac{\partial^2\Phi^{(1)}(\mathbf{G}\mathbf{t};\boldsymbol{\nu},\boldsymbol{\Psi})}{\partial\mathbf{t}\partial\mathbf{t}^{\mathrm{T}}}\Big|_{\mathbf{t}=\mathbf{0}} = \phi^{(1)}(0;\boldsymbol{\nu},\boldsymbol{\Psi})\boldsymbol{\Psi}^{-1}\boldsymbol{\nu}\mathbf{G}^{\mathrm{T}}\mathbf{G};$$

and when $p = 2$ we have

$$\frac{\partial \Phi^{(2)}(\mathbf{Gt};\boldsymbol{\nu},\boldsymbol{\Psi})}{\partial \mathbf{t}}\,|_{\mathbf{t}=\mathbf{0}} = \sum_{\ell=1}^{2} \Phi^{(1)}\Big(\mathbf{0};\boldsymbol{\nu}_{-\ell} - \boldsymbol{\Psi}_{-\ell,\ell}\boldsymbol{\Psi}_{\ell,\ell}^{-1}\boldsymbol{\nu}_{\ell}, \boldsymbol{\Psi}_{-\ell,-\ell} - \boldsymbol{\Psi}_{-\ell,\ell}\boldsymbol{\Psi}_{\ell,\ell}^{-1}\boldsymbol{\Psi}_{\ell,-\ell}\Big)$$
$$\phi^{(1)}(0;\boldsymbol{\nu}_{\ell},\boldsymbol{\Psi}_{\ell,\ell})\mathbf{G}_{\ell\cdot}^{\mathrm{T}},$$

$$\frac{\partial^2 \Phi^{(2)}(\mathbf{Gt};\boldsymbol{\nu},\boldsymbol{\Psi})}{\partial \mathbf{t}\partial \mathbf{t}^{\mathrm{T}}}\,|_{\mathbf{t}=\mathbf{0}} = \sum_{\ell=1}^{2} \left[\phi^{(1)}(0;\boldsymbol{\nu}^{\ell*},\boldsymbol{\Psi}^{\ell*})\phi^{(1)}(0;\boldsymbol{\nu}_{\ell},\boldsymbol{\Psi}_{\ell,\ell})\mathbf{G}_{\ell\cdot}^{\mathrm{T}}\mathbf{G}^{\ell*}\right]$$
$$+\sum_{\ell=1}^{2} \Phi^{(1)}\big(\mathbf{0};\boldsymbol{\nu}^{\ell*},\boldsymbol{\Psi}^{\ell*}\big)\phi^{(1)}(0;\boldsymbol{\nu}_{\ell},\boldsymbol{\Psi}_{\ell,\ell})\boldsymbol{\Psi}_{\ell,\ell}^{-1}\boldsymbol{\nu}_{\ell}\mathbf{G}_{\ell\cdot}^{\mathrm{T}}\mathbf{G}_{\ell\cdot}.$$

2. Conditional distribution of the random effects given observed data

Following Section 2.2 of the main text, the conditional distribution of the random effects $\mathbf{b}_i$, given the observed data $Y_{i1},\ldots,Y_{i,j-1},S_i = j-1,\mathbf{H}_{i,j-1}$, is

$$\begin{aligned}
& f(\mathbf{b}_i \mid Y_{i1},\ldots,Y_{i,j-1},S_i = j-1,\mathbf{H}_{i,j-1}) \\
\propto\ & f(\mathbf{b}_i;\boldsymbol{\theta})f(Y_{i1},\ldots,Y_{i,j-1} \mid \mathbf{H}_{i,j-1},\mathbf{b}_i;\boldsymbol{\theta})\lambda_{i,j-1}\prod_{l=1}^{j-2}(1-\lambda_{il}) \\
\propto\ & \exp\{-\frac{1}{2}\mathbf{b}_i^{\mathrm{T}}\boldsymbol{\Sigma}_b^{-1}\mathbf{b}_i - \frac{1}{2\sigma_\epsilon^2}\sum_{\ell=1}^{j-1}(y_{i\ell} - \mathbf{x}_{i\ell}^{\mathrm{T}}\boldsymbol{\beta} - \mathbf{z}_{i\ell}^{\mathrm{T}}\mathbf{b}_i)^2\}\Phi^{(j-1)}(\mathbf{A}_{j-1}\boldsymbol{\alpha} + \mathbf{B}_{j-1}\mathbf{b}_i;\mathbf{0},\mathbf{I}) \\
\propto\ & \exp\left[-\frac{1}{2}\left\{\mathbf{b}_i^{\mathrm{T}}\left(\boldsymbol{\Sigma}_b^{-1} + \frac{1}{\sigma_\epsilon^2}\sum_{\ell=1}^{j-1} z_{i\ell}z_{i\ell}^{\mathrm{T}}\right)\mathbf{b}_i - \frac{2}{\sigma_\epsilon^2}\sum_{\ell=1}^{j-1}(y_{i\ell} - \mathbf{x}_{i\ell}^{\mathrm{T}}\boldsymbol{\beta})\mathbf{z}_{i\ell}^{\mathrm{T}}\mathbf{b}_i\right\}\right] \\
& \Phi^{(j-1)}(\mathbf{A}_{j-1}\boldsymbol{\alpha} + \mathbf{B}_{j-1}\mathbf{b}_i;\mathbf{0},\mathbf{I}) \\
\propto\ & \phi^{(q)}(\mathbf{b}_i;\boldsymbol{\mu}_{b_i|\cdot},\boldsymbol{\Sigma}_{b_i|\cdot})\Phi^{(j-1)}(\mathbf{A}_{j-1}\boldsymbol{\alpha} + \mathbf{B}_{j-1}\mathbf{b}_i;\mathbf{0},\mathbf{I}),
\end{aligned}$$

where $\boldsymbol{\Sigma}_{b_i|\cdot} = (\boldsymbol{\Sigma}_b^{-1} + \frac{1}{\sigma_\epsilon^2}\sum_{\ell=1}^{j-1} z_{i\ell}z_{i\ell}^{\mathrm{T}})^{-1}$, $\boldsymbol{\mu}_{b_i|\cdot} = \frac{1}{\sigma_\epsilon^2}\boldsymbol{\Sigma}_{b_i|\cdot}\sum_{\ell=1}^{j-1}(y_{i\ell} - \mathbf{x}_{i\ell}^{\mathrm{T}}\boldsymbol{\beta})\mathbf{z}_{i\ell}$, $\mathbf{A}_{j-1} = \begin{pmatrix} \mathbf{x}_{S,i1}^{\mathrm{T}} \\ \vdots \\ \mathbf{x}_{S,i,j-2}^{\mathrm{T}} \\ -\mathbf{x}_{S,i,j-1}^{\mathrm{T}} \end{pmatrix}$, and $\mathbf{B}_{j-1} = \begin{pmatrix} \boldsymbol{\gamma}_1^{\mathrm{T}}\mathbf{W}_{i1} \\ \vdots \\ \boldsymbol{\gamma}_{j-2}^{\mathrm{T}}\mathbf{W}_{i,j-2} \\ -\boldsymbol{\gamma}_{j-1}^{\mathrm{T}}\mathbf{W}_{i,j-1} \end{pmatrix}$. Therefore, it is a multivariate skew-

normal distribution with density function

$$\begin{aligned} & \phi^{(q)}(\mathbf{b}_i; \boldsymbol{\mu}_{b_i|\cdot}, \boldsymbol{\Sigma}_{b_i|\cdot}) \frac{\Phi^{(j-1)}(\mathbf{A}_{j-1}\boldsymbol{\alpha} + \mathbf{B}_{j-1}\mathbf{b}_i; \mathbf{0}, \mathbf{I})}{\Phi^{(j-1)}(\mathbf{A}_{j-1}\boldsymbol{\alpha} + \mathbf{B}_{j-1}\boldsymbol{\mu}_{b_i|\cdot}; \mathbf{0}, \mathbf{I} + \mathbf{B}_{j-1}\boldsymbol{\Sigma}_{b_i|\cdot}\mathbf{B}_{j-1}^{\mathrm{T}})} \\ = \; & \phi^{(q)}(\mathbf{b}_i; \boldsymbol{\mu}_{b_i|\cdot}, \boldsymbol{\Sigma}_{b_i|\cdot}) \frac{\Phi^{(j-1)}(\mathbf{B}_{j-1}(\mathbf{b}_i - \boldsymbol{\mu}_{b_i|\cdot}); -\mathbf{A}_{j-1}\boldsymbol{\alpha} - \mathbf{B}_{j-1}\boldsymbol{\mu}_{b_i|\cdot}, \mathbf{I})}{\Phi^{(j-1)}\Big(\mathbf{0}; -\mathbf{A}_{j-1}\boldsymbol{\alpha} - \mathbf{B}_{j-1}\boldsymbol{\mu}_{b_i|\cdot}, \mathbf{I} + \mathbf{B}_{j-1}\boldsymbol{\Sigma}_{b_i|\cdot}\mathbf{B}_{j-1}^{\mathrm{T}}\Big)}, \end{aligned} \tag{2}$$

where from (1), $\boldsymbol{\mu} = \boldsymbol{\mu}_{b_i|\cdot}$, $\boldsymbol{\Sigma} = \boldsymbol{\Sigma}_{b_i|\cdot}$, $\mathbf{D} = \mathbf{B}_{j-1}$, $\boldsymbol{\nu} = -\mathbf{A}_{j-1}\boldsymbol{\alpha} - \mathbf{B}_{j-1}\boldsymbol{\mu}_{b_i|\cdot}$, and $\boldsymbol{\Delta} = \mathbf{I}$.

3. Default extrapolation distribution for Y_{ik} $(j \leqslant k \leqslant M)$ under the SPM

Recall that under the specified SPM, it is assumed that the missing outcomes after dropout at $S_i = j-1$ still follows the model $Y_{ik} = \mathbf{x}_{ik}^{\mathrm{T}}\boldsymbol{\beta} + \mathbf{z}_{ik}^{\mathrm{T}}\mathbf{b}_i + \epsilon_{ik}$ $(j \leqslant k \leqslant M)$.

As shown in Section 2, we have

$$\begin{aligned} & f(\mathbf{b}_i \mid Y_{i1}, \ldots, Y_{i,j-1}, S_i = j-1, \mathbf{H}_{i,j-1}) \\ = \; & \phi^{(q)}(\mathbf{b}_i; \boldsymbol{\mu}_{b_i|\cdot}, \boldsymbol{\Sigma}_{b_i|\cdot}) \frac{\Phi^{(j-1)}(\mathbf{A}_{j-1}\boldsymbol{\alpha} + \mathbf{B}_{j-1}\mathbf{b}_i; \mathbf{0}, \mathbf{I})}{\Phi^{(j-1)}(\mathbf{A}_{j-1}\boldsymbol{\alpha} + \mathbf{B}_{j-1}\boldsymbol{\mu}_{b_i|\cdot}; \mathbf{0}, \mathbf{I} + \mathbf{B}_{j-1}\boldsymbol{\Sigma}_{b_i|\cdot}\mathbf{B}_{j-1}^{\mathrm{T}})} \\ = \; & \phi^{(q)}(\mathbf{b}_i; \boldsymbol{\mu}_{b_i|\cdot}, \boldsymbol{\Sigma}_{b_i|\cdot}) \frac{\Phi^{(j-1)}\Big(\mathbf{B}_{j-1}(\mathbf{b}_i - \boldsymbol{\mu}_{b_i|\cdot}); -(\mathbf{A}_{j-1}\boldsymbol{\alpha} + \mathbf{B}_{j-1}\boldsymbol{\mu}_{b_i|\cdot}), \mathbf{I}\Big)}{\Phi^{(j-1)}\Big(\mathbf{0}; -\mathbf{A}_{j-1}\boldsymbol{\alpha} - \mathbf{B}_{j-1}\boldsymbol{\mu}_{b_i|\cdot}, \mathbf{I} + \mathbf{B}_{j-1}\boldsymbol{\Sigma}_{b_i|\cdot}\mathbf{B}_{j-1}^{\mathrm{T}}\Big)} \\ \triangleq \; & CSN_{q,j-1}(\boldsymbol{\mu}_{b_i|\cdot}, \boldsymbol{\Sigma}_{b_i|\cdot}, \mathbf{B}_{j-1}, -(\mathbf{A}_{j-1}\boldsymbol{\alpha} + \mathbf{B}_{j-1}\boldsymbol{\mu}_{b_i|\cdot}), \mathbf{I}). \end{aligned}$$

According to **Remark 1** in González-Farías et al. (2004), the linear combination of the random effects $\mathbf{z}_{ik}^{\mathrm{T}}\mathbf{b}_i$ also follows a skew-normal distribution

$$\mathbf{z}_{ik}^{\mathrm{T}}\mathbf{b}_i \sim CSN_{1,j-1}(\mu_{1ik|\cdot}, \sigma^2_{1ik|\cdot}, \mathbf{D}_{1ik|\cdot}, \boldsymbol{\nu}_{1ik|\cdot}, \boldsymbol{\Delta}_{1ik|\cdot}),$$

where $\mu_{1ik|\cdot} = \mathbf{z}_{ik}^{\mathrm{T}}\boldsymbol{\mu}_{b_i|\cdot}$, $\sigma^2_{1ik|\cdot} = \mathbf{z}_{ik}^{\mathrm{T}}\boldsymbol{\Sigma}_{b_i|\cdot}\mathbf{z}_{ik}$, $\mathbf{D}_{1ik|\cdot} = \frac{1}{\sigma^2_{1ik|\cdot}}\mathbf{B}_{j-1}\boldsymbol{\Sigma}_{b_i|\cdot}\mathbf{z}_{ik}$, $\boldsymbol{\nu}_{1ik|\cdot} = -(\mathbf{A}_{j-1}\boldsymbol{\alpha} + \mathbf{B}_{j-1}\boldsymbol{\mu}_{b_i|\cdot})$, $\boldsymbol{\Delta}_{1ik|\cdot} = \mathbf{I} + \mathbf{B}_{j-1}\boldsymbol{\Sigma}_{b_i|\cdot}\mathbf{B}_{j-1}^{\mathrm{T}} - \frac{1}{\sigma^2_{1ik|\cdot}}\mathbf{B}_{j-1}\boldsymbol{\Sigma}_{b_i|\cdot}\mathbf{z}_{ik}\mathbf{z}_{ik}^{\mathrm{T}}\boldsymbol{\Sigma}_{b_i|\cdot}\mathbf{B}_{j-1}^{\mathrm{T}}$, with the density function

$$\phi^{(1)}(z; \mu_{1ik|\cdot}, \sigma^2_{1ik|\cdot}) \frac{\Phi^{(j-1)}\Big(\mathbf{D}_{1ik|\cdot}(z - \mu_{1ik|\cdot}); \boldsymbol{\nu}_{1ik|\cdot}, \boldsymbol{\Delta}_{1ik|\cdot}\Big)}{\Phi^{(j-1)}\Big(\mathbf{0}; \boldsymbol{\nu}_{1ik|\cdot}, \boldsymbol{\Delta}_{1ik|\cdot} + \sigma^2_{1ik|\cdot}\mathbf{D}_{1ik|\cdot}\mathbf{D}_{1ik|\cdot}^{\mathrm{T}}\Big)}.$$

Under the specified SPM in the main text, we have $\mathbf{x}_{ik}^{\mathrm{T}}\boldsymbol{\beta} + \epsilon_{ik} \sim N(\mathbf{x}_{ik}^{\mathrm{T}}\boldsymbol{\beta}, \sigma^2_\epsilon)$, which is a special case of the skew-normal distribution, and is independent of $\mathbf{z}_{ik}^{\mathrm{T}}\mathbf{b}_i$ given the covariates.

Therefore following **Theorem 4** in González-Farías et al. (2004), the conditional distribution for the missing data $Y_{ik} = \mathbf{x}_{ik}^{\mathrm{T}}\boldsymbol{\beta} + \mathbf{z}_{ik}^{\mathrm{T}}\mathbf{b}_i + \epsilon_{ik}$ given $Y_{i1}, \ldots, Y_{i,j-1}, S_i = j-1, \mathbf{H}_{i,j-1}, \mathbf{x}_{ik}, \mathbf{z}_{ik}$ (i.e., the default extrapolation distribution under the SPM) is also skew-normally distributed.

The moment generating function is

$$
\begin{aligned}
& M_{Y_{ik}|Y_{i1},\ldots,Y_{i,j-1},S_i=j-1,\mathbf{H}_{i,j-1},\mathbf{x}_{ik},\mathbf{z}_{ik}}(t) \\
= \;& M_{\mathbf{x}_{ik}^{\mathrm{T}}\boldsymbol{\beta}+\epsilon_{ik}}(t) M_{\mathbf{z}_{ik}^{\mathrm{T}}\mathbf{b}_i|Y_{i1},\ldots,Y_{i,j-1},S_i=j-1,\mathbf{H}_{i,j-1}}(t) \\
= \;& \exp\left(\mathbf{x}_{ik}^{\mathrm{T}}\boldsymbol{\beta}t + \frac{1}{2}\sigma_\epsilon^2 t^2\right) \exp\left\{\mu_{1ik|\cdot}t + \frac{1}{2}\sigma_{1ik|\cdot}^2 t^2\right\} \\
& \frac{\Phi^{(j-1)}(\mathbf{D}_{1ik|\cdot}\sigma_{1ik|\cdot}^2 t; \boldsymbol{\nu}_{1ik|\cdot}, \boldsymbol{\Delta}_{1ik|\cdot} + \sigma_{1ik|\cdot}^2 \mathbf{D}_{1ik|\cdot}\mathbf{D}_{1ik|\cdot}^{\mathrm{T}})}{\Phi^{(j-1)}(\mathbf{0}; \boldsymbol{\nu}_{1ik|\cdot}, \boldsymbol{\Delta}_{1ik|\cdot} + \sigma_{1ik|\cdot}^2 \mathbf{D}_{1ik|\cdot}\mathbf{D}_{1ik|\cdot}^{\mathrm{T}}} \\
= \;& \exp\left\{(\mathbf{x}_{ik}^{\mathrm{T}}\boldsymbol{\beta} + \mu_{1ik|\cdot})t + \frac{1}{2}(\sigma_\epsilon^2 + \sigma_{1ik|\cdot}^2)t^2\right\} \\
& \frac{\Phi^{(j-1)}(\mathbf{D}_{1ik|\cdot}\sigma_{1ik|\cdot}^2 t; \boldsymbol{\nu}_{1ik|\cdot}, \boldsymbol{\Delta}_{1ik|\cdot} + \sigma_{1ik|\cdot}^2 \mathbf{D}_{1ik|\cdot}\mathbf{D}_{1ik|\cdot}^{\mathrm{T}})}{\Phi^{(j-1)}(\mathbf{0}; \boldsymbol{\nu}_{1ik|\cdot}, \boldsymbol{\Delta}_{1ik|\cdot} + \sigma_{1ik|\cdot}^2 \mathbf{D}_{1ik|\cdot}\mathbf{D}_{1ik|\cdot}^{\mathrm{T}}} \\
= \;& \exp\left(\mu_{2ik|\cdot}t + \frac{1}{2}\sigma_{2ik|\cdot}^2 t^2\right) \frac{\Phi^{(j-1)}(\mathbf{D}_{2ik|\cdot}\sigma_{2ik|\cdot}^2 t; \boldsymbol{\nu}_{2ik|\cdot}, \boldsymbol{\Delta}_{2ik|\cdot} + \sigma_{2ik|\cdot}^2 \mathbf{D}_{2ik|\cdot}\mathbf{D}_{2ik|\cdot}^{\mathrm{T}})}{\Phi^{(j-1)}(\mathbf{0}; \boldsymbol{\nu}_{2ik|\cdot}, \boldsymbol{\Delta}_{2ik|\cdot} + \sigma_{2ik|\cdot}^2 \mathbf{D}_{2ik|\cdot}\mathbf{D}_{2ik|\cdot}^{\mathrm{T}})}
\end{aligned}
$$

where $\mu_{2ik|\cdot} = \mathbf{x}_{ik}^{\mathrm{T}}\boldsymbol{\beta} + \mu_{1ik|\cdot}$, $\sigma_{2ik|\cdot}^2 = \sigma_\epsilon^2 + \sigma_{1ik|\cdot}^2$, $\mathbf{D}_{2ik|\cdot} = \frac{\sigma_{1ik|\cdot}^2}{\sigma_\epsilon^2+\sigma_{1ik|\cdot}^2}\mathbf{D}_{1ik|\cdot}$, $\boldsymbol{\nu}_{2ik|\cdot} = \boldsymbol{\nu}_{1ik|\cdot}$, $\boldsymbol{\Delta}_{2ik|\cdot} = \boldsymbol{\Delta}_{1ik|\cdot} + \sigma_{1ik|\cdot}^2\mathbf{D}_{1ik|\cdot}\mathbf{D}_{1ik|\cdot}^{\mathrm{T}} - \frac{\sigma_{1ik|\cdot}^4}{\sigma_\epsilon^2+\sigma_{1ik|\cdot}^2}\mathbf{D}_{1ik|\cdot}\mathbf{D}_{1ik|\cdot}^{\mathrm{T}}$. Thus it follows that the default extrapolation distribution for Y_{ik} under the specified SPM is a skew-normal distribution with the density function

$$
\begin{aligned}
& f_{Y_{ik}|Y_{i1},\ldots,Y_{i,j-1},S_i=j-1,\mathbf{H}_{i,j-1}}(y_{ik}) \\
= \;& \phi^{(1)}(y_{ik}; \mu_{2ik|\cdot}, \sigma_{2ik|\cdot}^2) \frac{\Phi^{(j-1)}(D_{2ik|\cdot}(y_{ik} - \mu_{2ik|\cdot}); \boldsymbol{\nu}_{2ik|\cdot}, \boldsymbol{\Delta}_{2ik|\cdot})}{\Phi^{(j-1)}(\mathbf{0}; \boldsymbol{\nu}_{2ik|\cdot}, \boldsymbol{\Delta}_{2ik|\cdot} + \sigma_{2ik|\cdot}^2 \mathbf{D}_{2ik|\cdot}\mathbf{D}_{2ik|\cdot}^{\mathrm{T}})}.
\end{aligned}
$$

4. Steps for model assessment

The steps to implement the model assessment procedure in Section 3.3 of the main text are:

(1) For the ith patient, sample the complete longitudinal outcome vector, $\mathbf{Y}_i^{rep}$, from the specified longitudinal model in (8) of the main text, given the current posterior samples and the patient's covariate values.

(2) For the ith patient, sample a replicated dropout time, S_i^{rep}, from the specified dropout model in (9) of the main text, given the current posterior samples and the patient's covariate values.

(3) If $S_i^{rep} = j - 1 < 12$, truncate $\mathbf{Y}_i^{rep}$ at visit $j - 1$ to obtain the replicates of the observed longitudinal data $\mathbf{Y}_i^{o,rep}$.

(4) Repeat 1-3 for $N = 827$ HERS patients and compute $\sum_{i=1}^{827}(\mathbf{Y}_i^{o,rep} - \boldsymbol{\mu}_i^o)^{\mathrm{T}}\mathbf{V}{}_i^{o-1}(\mathbf{Y}_i^{o,rep} - \boldsymbol{\mu}_i^o)/n_i^{rep}$, where $\boldsymbol{\mu}_i^o = \mathrm{E}(\mathbf{Y}_i^{o,rep} \mid S_i^{rep} = j-1, \mathbf{H}_{i,j-1})$, $\mathbf{V}_i^o = \mathrm{Var}(\mathbf{Y}_i^{o,rep} \mid S_i^{rep} = j - 1, \mathbf{H}_{i,j-1})$, $\mathbf{H}_{i,j-1}$ is the collection of the covariates in (8) and (9) of the main text up to visit $j - 1$, and n_i^{rep} is the number of observations in $\mathbf{Y}_i^{o,rep}$. Details for computing $\boldsymbol{\mu}_i^o$ and $\mathbf{V}_i^o$ can be found in Section 5.

(5) Compute the χ^2 discrepancy for the observed HERS data, similarly to Step 4 by replacing $\mathbf{Y}_i^{o,rep}$ with $\mathbf{Y}_i^o$.

(6) Repeat Steps 1-5 for each posterior sample of the model parameters and compute the posterior predictive probability that the replicated χ^2 statistic is larger than the observed χ^2 statistic.

Because computing $\boldsymbol{\mu}_i^o$ and $\mathbf{V}_i^o$ involves numerous calculations of multivariate normal probabilities, we only use 1024 (out of 9000) posterior samples and parallelize the calculation of the χ^2 discrepancy statistics on 32 cores. The posterior probability that the χ^2 statistic is larger than the observed χ^2 statistic is 0.212, which does not indicate lack of fit of our SPM to the observed HERS data.

For model comparison, Deviance Information Criterion (DIC) (Spiegelhalter et al., 2002) based on observed data likelihood can be used (Wang and Daniels, 2011). This requires integrating out the random effects to obtain the observed data likelihood, which is computationally intensive for the SPM. Note that WinBUGS provides the DIC conditional on random effects; therefore the DIC from WinBUGS is not appropriate for model comparison.

In addition, because the LMM does not explicitly model the dropout process, its DIC based on observed data likelihood can not be directly compared with that from the SPM. But we could compare the values of DIC for the SPM and PMM. More discussion about model comparison with informative dropout can be found in Daniels et al. (2012).

5. Conditional distribution of the random effects given the dropout time and covariates

For computing the χ^2 discrepancy statistics in the model assessment procedure in Section 4, we need to compute $\mathrm{E}(\mathbf{Y}_i^{o,rep} \mid S_i^{rep} = j-1, \mathbf{H}_{i,j-1})$ and $\mathrm{Var}(\mathbf{Y}_i^{o,rep} \mid S_i^{rep} = j-1, \mathbf{H}_{i,j-1})$.

We first derive the conditional distribution of the random effect $\mathbf{b}_i$ given the dropout time $S_i^{rep} = j-1$ and the covariates $\mathbf{H}_{i,j-1}$ and obtain its mean and variance. Then writing $\mathbf{Y}_i^{o,rep} = \mathbf{X}_i^o\boldsymbol{\beta} + \mathbf{Z}_i^o\mathbf{b}_i + \boldsymbol{\epsilon}_i^{o,rep}$, where $\mathbf{X}_i^o = (\mathbf{x}_{i1}, \ldots, \mathbf{x}_{i,j-1})^{\mathrm{T}}$ and $\mathbf{Z}_i^o = (\mathbf{z}_{i1}, \ldots, \mathbf{z}_{i,j-1})^{\mathrm{T}}$, and $\boldsymbol{\epsilon}_i^{o,rep} = (\epsilon_{i1}^{rep}, \ldots, \epsilon_{i,j-1}^{rep})^{\mathrm{T}}$, we can easily obtain $\mathrm{E}(\mathbf{Y}_i^{o,rep} \mid S_i^{rep} = j-1, \mathbf{H}_{i,j-1}) = \mathbf{X}_i^o\boldsymbol{\beta} + \mathbf{Z}_i^o\mathrm{E}(\mathbf{b}_i \mid S_i^{rep} = j-1, \mathbf{H}_{i,j-1})$ and $\mathrm{Var}(\mathbf{Y}_i^{o,rep} \mid S_i^{rep} = j-1, \mathbf{H}_{i,j-1}) = \mathbf{Z}_i^o\mathrm{Var}(\mathbf{b}_i \mid S_i^{rep} = j-1, \mathbf{H}_{i,j-1})(\mathbf{Z}_i^o)^{\mathrm{T}} + \sigma_\epsilon^2\mathbf{I}$.

Since

$$
\begin{aligned}
& f(\mathbf{b}_i \mid S_i^{rep} = j-1, \mathbf{H}_{i,j-1}) \\
\propto\ & f(S_i^{rep} = j-1 \mid \mathbf{b}_i, \mathbf{H}_{i,j-1}) f(\mathbf{b}_i \mid \mathbf{H}_{i,j-1}) \\
\propto\ & \lambda_{i,j-1} \prod_{l=1}^{j-2} (1-\lambda_{il}) \phi^{(q)}(\mathbf{b}_i; \mathbf{0}, \boldsymbol{\Sigma}_b) \\
\propto\ & \Phi^{(j-1)}(\mathbf{A}_{j-1}\boldsymbol{\alpha} + \mathbf{B}_{j-1}\mathbf{b}_i; \mathbf{0}, \mathbf{I}) \phi^{(q)}(\mathbf{b}_i; \mathbf{0}, \boldsymbol{\Sigma}_b),
\end{aligned}
$$

it follows that the conditional distribution of $\mathbf{b}_i$ given $S_i^{rep} = j-1, \mathbf{H}_{i,j-1}$ is a multivariate skew-normal distribution with the density function

$$
f(\mathbf{b}_i \mid S_i = j-1, \mathbf{H}_{i,j-1}) = \phi^{(q)}(\mathbf{b}_i; \mathbf{0}, \boldsymbol{\Sigma}_b) \frac{\Phi^{(j-1)}(\mathbf{B}_{j-1}\mathbf{b}_i; -\mathbf{A}_{j-1}\boldsymbol{\alpha}, \mathbf{I})}{\Phi^{(j-1)}(\mathbf{0}; -\mathbf{A}_{j-1}\boldsymbol{\alpha}, \mathbf{I} + \mathbf{B}_{j-1}\boldsymbol{\Sigma}_b\mathbf{B}_{j-1}^{\mathrm{T}})}.
$$

6. Results from a pattern mixture model for the HERS data

Pattern mixture models (PMMs) are an alternative approach to dealing with informative dropout and can also provide transparent sensitivity analyses. Discussion about advantages and disadvantages of PMMs can be found in Chapter 8 of Daniels and Hogan (2008). Hogan et al. (2004) provided an analysis of the HERS data using a PMM. Here we follow their approach and compare the results from the PMM with those from the SPM. The observed dropout times are first combined to form three dropout pattern groups: patients whose final visits were between visit 1 and visit 5; patients whose final visits were between visit 6 and visit 10; patients whose final visits were visit 11 or visit 12 (completers). Discussion of this choice of grouping can be found in Hogan et al. (2004). A linear mixed model is then specified such that each dropout pattern group has unique regression coefficients for the fixed effects. Specifically, let $G_i = g$ if the patient's final visit was in pattern g ($g = 1, 2, 3$). We assume that

$$Y_{ij} = \mathbf{x}_{ij}^{\mathrm{T}}\boldsymbol{\beta}^{g} + b_{i1} + b_{i2}j^{*} + \epsilon_{ij}, \tag{3}$$

where $\boldsymbol{\beta}^g$ is the vector of regression coefficients for the gth grouped pattern. The specifications for random effects, the error term and the covariates remain the same as in the longitudinal sub-model of the SPM described in Section 3.1 of the main text. Independent normal priors $N(0, 100)$ are assigned to $\boldsymbol{\beta}^g$. The prior specification for the rest of the parameters is the same as in the SPM.

Table 1 presents the posterior mean estimates and 95% credible intervals of the regression coefficients for each dropout pattern. All of the coefficients vary significantly across patterns. The estimated main effects of time are negative for patterns 1-2, but are close to zero for pattern 3. In particular, the magnitude of these effects decrease as the dropout time increases. To summarize the marginal effects of covariates unconditional on dropout patterns, we take

a weighted average of the conditional effects over the distribution of the dropout patterns:

$$\boldsymbol{\beta} = \sum_{g=1}^{3} \boldsymbol{\beta}^g \pi^g,$$

where π^g is the probability of being in pattern g. We assume that G_i follows a multinomial distribution with parameters π^1, π^2, π^3 that are given a Dirichlet prior. We then use the posterior samples of π^g and $\boldsymbol{\beta}^g$ to obtain the posterior samples of $\boldsymbol{\beta}$. Table 1 presents the posterior mean estimates and 95% credible intervals of $\boldsymbol{\beta}$. The estimated marginal covariate effects, especially the main effects, are similar in the PMM and SPM. As a result, the conclusions about the effects of baseline viral load levels and ART status on the rates of changes in mean CD4 counts are similar in both analyses.

Based on the fitted PMM, a sensitivity analysis can be done by extrapolating the mean longitudinal CD4 count profiles beyond dropout for those patients who had the same dropout times. Similar to the proposed sensitivity analysis approach, a piece-wise linear model can be used and sensitivity parameters can be specified to allow for the change of longitudinal profiles after dropout to be a function of the dropout time and also the observed covariates. Then the conditional longitudinal profiles are averaged over the dropout time distribution and marginal covariate effects can be summarized based on the marginal longitudinal profiles. Su and Hogan (2010) provided an example how this can be done in the HERS data analysis. An important distinction of the sensitivity analysis based on the PMM from that based on the SPM is that for the PMM the extrapolation is done at the population level for each unique dropout time. Therefore, summaries of marginal covariate effects can be done directly by averaging over the observed dropout time distribution, and do not have to rely on G-computation. However, this approach can be difficult to implement when there are many unique observed dropout times and data are sparse within each unique dropout time.

[Table 1 about here.]

7. Approximation for $\sigma_{b_{i2}}$ in G-computation

To speed up the G-computation for the HERS analysis, we approximate $\sigma_{b_{i2}}$ using the average estimated posterior standard deviations of the random slopes for all HERS patients within each of the 8 covariate groups defined by the baseline viral load level, ART status and HIV symptoms. Specifically, we first obtain empirical standard deviations of random slopes of the HERS patients in WinBUGS (based on 600 posterior samples of each random slope), and then average them within baseline covariate groups. Table 2 presents the approximated values $\widehat{\sigma}_{b_{i2}}$ for $\sigma_{b_{i2}}$ by the baseline covariate groups.

[Table 2 about here.]

We compare the results of marginal covariate effects when the exact and approximated values of $\sigma_{b_{i2}}$ are used in the G-computation of the sensitivity analysis for the HERS data. It appears that the point estimates of the marginal covariate effects are similar, but the 95% credible intervals are wider when the exact values of $\sigma_{b_{i2}}$ are used. For example, given that no ART was used and the number of HIV symptom at baseline was zero, the difference in the mean change of square root CD4 count from baseline to Visit 6 between the viral load 500-5k and 30k+ groups is 2.26 (95% CI=[0.85,3.70]) when the exact values of $\sigma_{b_{i2}}$ are used. The estimate of this covariate effect is 2.11 (95% CI=[0.76,3.37]) when the approximated values of $\sigma_{b_{i2}}$ are used. This is not surprising because in the approximation $\widehat{\sigma}_{b_{i2}}$ varies by baseline covariates only, but in the exact calculation $\sigma_{b_{i2}}$ is also a function of observed outcomes before dropout in additional to baseline covariates (thus the latter introduces more variation).

When the marginal covariate effects are more pronounced (e.g. the viral load effects), using the approximation or the exact values of $\sigma_{b_{i2}}$ would not make much difference in terms of statistical conclusions. But it could make slight differences for the covariate effects that are closer to zero because the 95% CI might cover zero when the exact values of $\sigma_{b_{i2}}$ are used. For example, given the viral load was 500-5k and the number of HIV symptom at baseline

was zero, the difference in the mean change of square root CD4 count from baseline to Visit 6 between baseline ART groups is 0.54 (95% CI=[-0.12,1.25]) when the exact values of $\sigma_{b_{i2}}$ are used. The estimate of this covariate effect is 0.63 (95% CI=[0.05,1.19]) when the approximated values of $\sigma_{b_{i2}}$ are used.

References

Arnold, B. C. (2009). Flexible univariate and multivariate models based on hidden truncation. *Journal of Statistical Planning and Inference* **139,** 3741–3749.

Daniels, M. J., Chatterjee, A. S., and Wang, C. (2012). Bayesian model selection for incomplete data using the posterior predictive distribution. *Biometrics* **68,** 1055–1063.

Daniels, M. J. and Hogan, J. W. (2008). *Missing Data in Longitudinal Studies: Strategies for Bayesian Modeling and Sensitivity Analysis.*, volume 101 of *Monographs on Statistics and Applied Probability.* Chapman & Hall/CRC, New York.

Flecher, C., Naveau, P., and Allard, D. (2009). Estimating the closed skew-normal distribution parameters using weighted moments. *Statistics and Probability Letters* **79,** 1977–1984.

González-Farías, G., Domínguez-Molina, A., and Gupta, A. K. (2004). Additive properties of skew normal random vectors. *Journal of Statistical Planning and Inference* **126,** 521–534.

Hogan, J. W., Roy, J., and Korkontzelou, C. (2004). Handling drop-out in longitudinal studies. *Statistics in Medicine* **23,** 1455–1497.

Spiegelhalter, D., Best, N., Carlin, B., and van der Linde, A. (2002). Bayesian measures of model complexity and fit. *Journal of the Royal Statistical Society. Series B* **64,** 583–639.

Su, L. and Hogan, J. W. (2010). Varying-coefficient models for longitudinal processes with continuous-time informative dropout. *Biostatistics* **11,** 93–110.

Wang, C. and Daniels, M. J. (2011). A note on MAR, identifying restrictions, model com-

parison, and sensitivity analysis in pattern mixture models with and without covariates for incomplete data. *Biometrics* **67,** 810–818.

Table 1
Posterior mean and 95% credible intervals of the model parameters in the PMM fitted to the HERS data.

	Marginal			Pattern 1			Pattern 2			Pattern 3		
	Mean	2.5%	97.5%	Mean	2.5%	97.5%	Mean	2.5%	97.5%	Mean	2.5%	97.5%
Intercept	-0.45	-0.67	-0.26	-0.59	-0.85	-0.32	-0.27	-0.81	0.24	-0.45	-0.76	-0.16
Baseline HIV viral load												
0-500	1.42	1.19	1.66	1.53	1.14	1.90	1.48	0.87	2.10	1.36	1.04	1.68
500-5k	0.91	0.70	1.13	0.92	0.61	1.22	0.66	0.13	1.25	0.97	0.67	1.30
5k-30k	0.43	0.20	0.68	0.23	-0.10	0.56	0.47	-0.16	1.07	0.51	0.17	0.88
30k+ (reference)												
Baseline HIV symptoms	-0.03	-0.08	0.03	-0.05	-0.16	0.06	-0.10	-0.25	0.05	0.00	-0.07	0.07
ART at baseline	-0.64	-0.76	-0.53	-0.48	-0.70	-0.25	-0.76	-1.07	-0.46	-0.69	-0.83	-0.54
Time (visit)	-1.22	-1.61	-0.84	-3.54	-4.53	-2.54	-2.25	-3.05	-1.46	0.01	-0.37	0.40
Time*baseline viral load												
0-500	0.88	0.44	1.35	4.00	2.64	5.35	1.43	0.53	2.39	-0.57	-0.97	-0.16
500-5k	0.33	-0.05	0.73	1.70	0.62	2.76	1.25	0.40	2.11	-0.48	-0.89	-0.07
5k-30k	0.20	-0.20	0.62	0.59	-0.49	1.69	0.70	-0.17	1.58	-0.09	-0.55	0.36
30k+ (reference)												
Time*baseline HIV symptoms	-0.02	-0.14	0.09	0.06	-0.33	0.45	0.03	-0.19	0.25	-0.07	-0.16	0.02
Time*ART at baseline	0.32	0.09	0.56	0.37	-0.40	1.15	-0.03	-0.50	0.44	0.40	0.22	0.59
corr(b_{i1}, b_{i2})	-0.31	-0.39	-0.23									
var(b_{i1})	0.55	0.49	0.61									
var(b_{i2})	0.91	0.79	1.05									
σ_ϵ^2	0.15	0.14	0.15									
Pr$(G_i = 1)$	0.25	0.22	0.28									
Pr$(G_i = 2)$	0.15	0.13	0.19									
Pr$(G_i = 3)$	0.60	0.56	0.63									

Table 2
Approximated values for $\sigma_{b_{i2}}$ by the baseline covariate groups

HIV symptoms	Viral load group	ART	$\widehat{\sigma}_{b_{i2}}$
0	0-500	No	0.48
0	0-500	Yes	0.52
0	500-5k	No	0.52
0	500-5k	Yes	0.49
0	5k-30k	No	0.61
0	5k-30k	Yes	0.62
0	30k+	No	0.69
0	30k+	Yes	0.79
